# Diplomatic monocultures in public health diplomacy: a narrative review on conference equity, participation and visibility

**DOI:** 10.3389/phrs.2026.1609708

**Published:** 2026-06-10

**Authors:** Martin Ernst, Lars Münter, Adam Skali, Eva Turk

**Affiliations:** 1 Center for Digital Health and Social Innovation, University of Applied Sciences St. Pölten, St. Pölten, Austria; 2 Nordic Wellbeing Academy, Birkeroed, Denmark; 3 European Health Futures Forum, Edergole/Dromahair, Ireland; 4 CIeS e-Health Innovation Center, University Mohammed V, Rabat, Morocco; 5 Institute for Human Centered Health Innovation (IHCHI), Basel, Switzerland

**Keywords:** capacity building, conferences, equity, global health governance, infodemic

## Abstract

**Objectives:**

Public health diplomacy increasingly unfolds under polycrisis conditions shaped by pandemics, conflict, demographic change, climate-related shocks, governance turbulence, and the infodemic. This narrative review examines conference equity, participation, and visibility as governance-relevant mechanisms within public health diplomacy.

**Methods:**

We conducted a narrative synthesis of recent literature on global health governance, public health diplomacy, conference participation, diversity and inclusion, digital and hybrid convening, and equity-oriented capacity building.

**Results:**

The synthesis indicates that participation and visibility gaps across gender, geography, country income context, career stage, language, mobility, and institutional resources can shape whose expertise is recognized, which agendas become prominent, and which coalitions form. We conceptualize these patterns as “diplomatic monocultures” that may narrow policy imagination, weaken legitimacy, and constrain capacity building. The review further identifies digital and hybrid formats as potential equity mechanisms only when designed to support comparable visibility, interaction, and influence.

**Conclusion:**

Conference equity should be treated as a public health diplomacy lever. We propose a multi-level roadmap for organizers, institutions, funders, and governance actors to strengthen equitable participation and more context-responsive global health governance.

## Introduction

### From crisis disruption to conference equity as diplomatic infrastructure

Public health diplomacy (PHD) increasingly unfolds in a polycrisis context. Pandemics, climate-related shocks, armed conflicts, humanitarian emergencies, demographic change, misinformation dynamics, and geopolitical realignment place health systems and governance institutions under simultaneous pressure [1–4]. Recent turbulence around the World Health Organization (WHO), including the announced withdrawal of the United States, illustrates how such disruptions can destabilize coordination structures while opening contested windows in which resources, institutional roles, and agenda-setting authority may be renegotiated [5–7]. In such moments, who is present, who is visible, and whose expertise is treated as credible becomes part of how public health priorities are debated, legitimized, and redistributed.

This review takes that observation as its starting point. PHD is often associated with formal diplomatic arenas, including intergovernmental negotiations, ministries, health attachés, and multilateral institutions. Yet much of the relational and epistemic work that enables diplomacy takes place before, around, and beyond formal negotiation rooms. Conferences, policy fora, expert meetings, and multilateral convening spaces are often formally organized structures with explicit programmes, procedures, and institutional goals. Yet within and around these formal settings, actors define problems, build coalitions, signal expertise, diffuse norms, and develop the relational trust required for later coordination. We therefore use the term “informal diplomatic infrastructure” not to suggest that conferences are informal in their organization, but to describe the informal diplomatic functions they enable: the networking, agenda-shaping, recognition, and coalition-building processes through which formal diplomacy is prepared, supported, and sometimes redirected [8–10]. Our aim is not to bypass or dismantle formal diplomatic structures, but to raise awareness of the cultures, routines, and participation norms surrounding them, because these influence whose voices are heard and whose perspectives become embedded in PHD.

The organising logic of this narrative review is that inequities in participation and visibility within convening spaces can become inequities in diplomatic influence. Participation determines who gains access to the rooms, platforms, and networks where public health priorities are discussed; visibility determines whose perspectives are publicly recognized as authoritative through keynotes, panel roles, session leadership, authorship, and other prestige signals. When these processes repeatedly privilege actors from well-resourced institutions, high-income settings, dominant language contexts, or established professional networks, conferences may contribute to “diplomatic monocultures”: recurring patterns in which a narrow range of actors and assumptions structures policy imagination.

Conversely, equitable participation and visibility can help transform convening spaces into more diverse diplomatic ecosystems. This matters especially during governance disruptions, when institutional arrangements are unsettled and influence may be redistributed. Redistribution windows navigated through narrow convening structures risk reproducing existing asymmetries; equity-oriented convening infrastructures may instead strengthen legitimacy, trust, capacity building, and context-responsive policy development.

**FIGURE 1:**
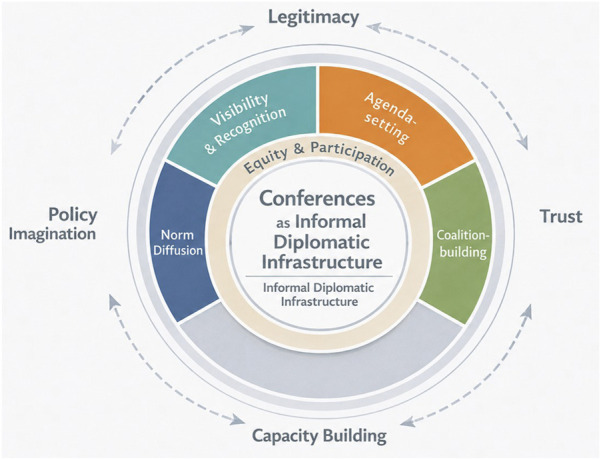
Conferences as informal diplomatic infrastructure. The figure depicts four conference-level mechanisms, Visibility & Recognition, Agenda-setting, Coalition-building, and Norm Diffusion, through which participation and visibility shape diplomacy-relevant outcomes (Legitimacy, Trust, Capacity Building, and Policy Imagination) (Austria, 2026).

**FIGURE 2:**
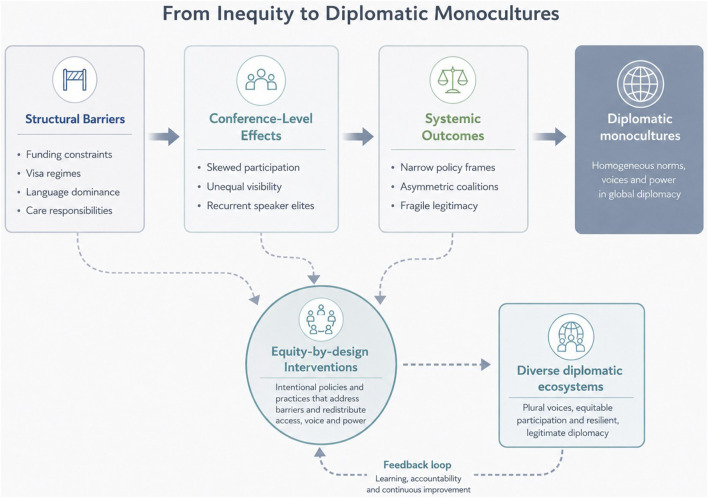
From inequity to diplomatic monocultures. A pathway model showing how structural barriers produce conference-level participation and visibility effects, which in turn generate systemic outcomes that reinforce diplomatic monocultures; equity-by-design interventions can redirect the pathway toward more diverse diplomatic ecosystems (Austria, 2026).

This organising logic structures the review in three steps. First, we conceptualize conferences as informal diplomatic infrastructure and outline how participation and visibility connect to recognition, agenda-setting, coalition-building, and norm diffusion (Figure 1). Second, we examine how inequities in access and recognition can form a pathway through which structural barriers translate into diplomatic monocultures (Figure 2). Third, we develop a multi-level roadmap in which conference organizers, institutions and funders, and global governance actors each hold levers for moving from *ad hoc* inclusion toward equity-by-design (Table 1).

**TABLE 1:** Multi-level roadmap for equitable conference governance (Austria, 2026).

Level	Intervention domain	Equity problem addressed	Concrete recommendations	Supporting sources
A. Conference organizers and professional societies	Transparent selection and accountability	Conference visibility is shaped by discretionary decisions about abstracts, invited sessions, keynotes, chairs, panels, and awards. Inequity can persist even when overall speaker diversity improves	Publish explicit criteria for invited roles; track and report speaker/chair composition by gender, geography, and income context; monitor high-status roles separately, including keynotes, plenaries, flagship panels, and chairs; create annual equity dashboards for recurrent conferences	[11–13]
A. Conference organizers and professional societies	Equity-by-design in programming	Agenda-setting roles may be concentrated among a narrow set of institutions, countries, and senior actors, reinforcing “diplomatic monocultures.”	Rotate intellectual leadership across regions; avoid repeated reliance on the same institutions and countries; include LMIC and high-burden-region expertise in plenaries and “state of the field” sessions; use subsidiarity-informed formats where local and regional priorities feed into global plenaries; apply design-oriented approaches to digital development where relevant [14]	[11, 12, 14, 15]
A. Conference organizers and professional societies	Mobility and access support	Costs, visa regimes, travel logistics, and opaque mobility processes disproportionately constrain LMIC and underrepresented participants	Pair acceptance decisions with early visa documentation; offer travel grants with early timelines and, where possible, pre-paid travel; provide visa support letters and administrative assistance; choose visa-accessible locations; treat mobility barriers as structural conference-design issues rather than individual problems	[11, 16, 17]
A. Conference organizers and professional societies	Hybrid parity and dual-format design	Hybrid access can widen attendance but relegate remote participants to lower visibility, weaker interaction, and limited networking	Treat remote participation as a first-class format rather than a streamed add-on; create mirrored plenary moments, shared decision points, and cross-format networking; rotate time zones; offer asynchronous access to key sessions; provide structured virtual networking, moderated Q&A, and remote chair/speaker roles	[18–21]
A. Conference organizers and professional societies	Facilitation and visibility	Representation does not guarantee influence; Q&A, moderation, and session design shape who is heard and recognized	Use moderated Q&A queues, text-based and verbal question options, structured small-group discussion feeding into plenaries, clear rules for respectful contestation, and active facilitation to prevent dominance effects; ensure remote participants can intervene visibly and not only observe	[20, 22, 23]
A. Conference organizers and professional societies	Institutionalized equity governance	Equity initiatives often depend on temporary enthusiasm and may disappear when conferences return to default in-person norms	Embed equity responsibilities in conference bylaws, programme committee mandates, standing diversity rules, and reporting procedures; include representation requirements for programme committees and invited roles; maintain institutional memory across annual conference cycles	[13, 18–20]
B. Institutions and funders	Dedicated participation funding with equity conditions	Participation funding is often fragmented, discretionary, and focused on attendance rather than visibility or influence	Create dedicated equitable-participation funds prioritizing LMIC participation, gender equity, underrepresented regions, and early-career actors; require funded conferences to report not only attendance but visible roles, including chairs, invited sessions, plenaries, and hybrid participation standards	[11, 20, 21]
B. Institutions and funders	Protected time and workload realism	Practitioners and researchers in under-resourced settings may lack protected time for conference participation, leadership service, and networking	Recognize conference leadership, dissemination, and public health diplomacy engagement as legitimate work outputs; include them in workload models and performance reviews; protect time for preparation, attendance, follow-up, and network-building	[11, 17, 24, 25]
B. Institutions and funders	Mobility infrastructure	High-visibility participation can fail because mobility support is late, informal, or institutionally uneven	Provide standardized visa and mobility assistance; centralize administrative support; fund travel early enough for visa processing; reduce hidden costs through pre-payment, per diem support, and flexible reimbursement policies	[11, 16, 17]
B. Institutions and funders	Equitable representation in consortium outputs	Multi-partner projects may reproduce hierarchy when the same institutions become default speakers, chairs, and representatives	Require conference dissemination equity plans in funded consortia; rotate speaking roles; include LMIC partners in high-visibility sessions; support co-chaired panels and co-led presentations; align conference visibility with partnership equity principles	[25, 26]
C. Governance actors and multi-stakeholder partnerships	Formalized inclusion pathways	Governance-adjacent convenings shape legitimacy, trust, and agenda-setting, but participation may remain concentrated among already powerful actors	Treat inclusion as legitimacy infrastructure; ensure representation from high-burden regions and LMIC actors in agenda-setting committees, plenaries, synthesis sessions, and negotiation-adjacent fora; involve underrepresented actors as co-architects, not only attendees	[12, 15, 27]
C. Governance actors and multi-stakeholder partnerships	Capacity-building and public health diplomacy pipeline	Conferences can build diplomacy competencies, but only if access to training, negotiation, and coalition-building spaces is equitable	Create diplomacy practice tracks, including negotiation workshops, cross-sector simulations, policy labs, consensus-building sessions, and mentorship formats; link travel and hybrid access support to competency development in negotiation, facilitation, communication, systems thinking, and coalition-building	[9, 10, 24, 28, 29]
C. Governance actors and multi-stakeholder partnerships	Safeguards during governance turbulence	Funding shocks, membership changes, treaty negotiations, and institutional crises can redistribute influence, but may also re-entrench existing asymmetries	Use moments of institutional change to broaden representation in agenda-setting fora; apply equity and subsidiarity principles; include local and regional expertise in defining priorities; prevent crisis convenings from defaulting to the same narrow set of high-visibility actors	[12, 15, 27]

Accordingly, this narrative review examines conference and convening equity as a governance-relevant mechanism within PHD rather than as a peripheral issue of academic fairness alone. Narrative synthesis was chosen because the relevant literature spans heterogeneous fields and study types, requiring conceptual integration rather than quantitative effect estimation [30]. The review is guided by three questions:

1. How can conferences and multilateral convening spaces be understood as informal diplomatic infrastructure within public health diplomacy?
2. Through which mechanisms do inequities in participation and visibility contribute to diplomatic monocultures?
3. Which multi-level measures can strengthen equitable participation and visibility as leverage points for legitimacy, trust, capacity building, and more context-responsive global health governance?

By foregrounding this organising logic, the review argues that conference design is also a question of how public health diplomacy distributes attention, authority, and the capacity to shape collective action.

## Methods

### Approach: narrative review and thematic synthesis

We conducted a narrative review to integrate evidence and conceptual arguments across heterogeneous literatures spanning global health governance, public health diplomacy, conference participation research, diversity and inclusion scholarship, digital and hybrid convening, and equity-oriented capacity building [11, 25, 26, 31]. A narrative review design was chosen because the aim was not to estimate pooled effects or to answer a narrowly reproducible intervention question, but to develop an organising conceptual synthesis across fields that use different study designs, evidentiary standards, and terminologies. Narrative synthesis was therefore considered appropriate because relevant contributions vary substantially in design, including bibliometric analyses, observational studies of speaker line-ups, policy commentaries, qualitative accounts, and program evaluations, and because the aim here is theory-informed integration rather than quantitative effect estimation [30]. The manuscript identifies the conceptual starting point of the review, namely that conference and convening equity can be understood as part of the informal diplomatic infrastructure of public health diplomacy.

The literature base, only including literature since 2020, was identified through iterative searches, targeted screening of reference lists in key papers, and purposive inclusion of sources that contributed directly to the review’s conceptual domains. We prioritized publications addressing participation, visibility, and representation in global health and public health-related conferences, with attention to gender, geography, career stage, and country income context, and included work linking convening spaces to networks, capacity building, and governance processes [11, 25, 26]. Additional sources were included when they provided conceptual frameworks relevant to public health diplomacy, such as informal diplomacy, legitimacy, coalition-building, systems thinking, infodemic governance, digital/hybrid convening, or practical interventions aimed at improving equitable participation [3, 4, 8–10, 29, 32, 33].

Because this was a narrative rather than a systematic or scoping review, we did not apply a formal database-based screening protocol, risk-of-bias assessment, or quantitative inter-coder reliability procedure. Instead, included sources were synthesized thematically and mapped onto a conceptual pathway linking participation and visibility in convening spaces to public health diplomacy mechanisms and outcomes. To increase transparency, Supplementary Table A1 provides a descriptive thematic mapping of the included sources and indicates which thematic domains each source informed. This appendix is intended to make the narrative synthesis more traceable without implying that the review followed the methodological procedures of a systematic review.

The main themes were not derived from a formal *a priori* coding scheme in the sense of systematic qualitative evidence synthesis. Rather, they were defined through an iterative, theory-informed thematic mapping process. Initial sensitising concepts were derived from the review questions and from the public health diplomacy literature, particularly the distinction between formal and informal diplomacy, and the relevance of legitimacy, trust, coalition-building, communication, and capacity building for diplomatic practice [3, 4, 8–10, 24, 28]. These concepts were then refined through engagement with the conference equity and inclusion literature, which highlighted recurring dimensions of participation, visibility, representation, access barriers, gender, geography, country income context, career stage, and organizational inclusion climates [11, 25, 26, 31, 32, 34–36]. Finally, sources on digital/hybrid convening, mobility barriers, infodemic governance, and systems thinking were used to specify the mechanisms and intervention levels reflected in the conceptual pathway and roadmap [12, 16–21, 29, 33, 37]. Thus, the thematic structure was developed as an interpretive framework rather than as a formal coding scheme: sources were mapped according to the actors, barriers, mechanisms, and diplomacy-relevant outcomes they addressed.

### Conceptual framing: how participation becomes diplomatic power

#### Conferences as sites of informal diplomacy

In PHD, a substantial portion of influence emerges outside formal negotiation rooms. Informal diplomacy works through relationship-building, shared problem definitions, and coordination among diverse actors. Conferences provide repeated, structured opportunities for initiating or continuing such processes: they facilitate network formation, broker cross-sector communication, and enable the “pre-negotiation” work through which priorities and coalitions crystallize [8–10]. Participation is therefore not merely a logistical matter; it shapes access to the relational infrastructure of diplomacy.

#### Visibility, credibility, and agenda-setting

Visibility (e.g., keynote invitations, panel placements, session chairs, and prominent authorship) functions as a reputational signal. Reputational signals shape whose evidence is perceived as credible, whose interpretation of crises becomes dominant, and which solutions are treated as feasible or legitimate [11, 31, 35]. Where visibility is systematically uneven, the conference sphere can reinforce asymmetries of epistemic authority, an issue that becomes particularly consequential during crises, when rapid consensus-building is needed and narratives can become locked in early [1, 2, 4].

#### Equity as capability and systems leverage

Equity in participation can be conceptualized as capability: a function of resources (travel funds, time, institutional support), structural barriers (visa regimes, discrimination), and enabling infrastructures (hybrid access, language inclusion, caregiving support) [11, 25, 26]. From a systems-thinking perspective, conferences operate as leverage points because they concentrate gatekeeping mechanisms (selection, invitation, scheduling) that can either amplify exclusion or systematically counteract it [29]. If improved, conference equity can support broader governance goals: legitimacy, trust, diversity of policy options, and resilience of partnerships [8, 29, 36]. Figure 1 summarizes the conceptual framing developed in Sections *Roadmap: policy and practice options across levels*, *Outlook: from conference equity to resilient public health diplomacy*, *Limitations* by visualizing conferences as informal diplomatic infrastructure. The inner mechanism layer distinguishes four recurring functions of conferences, Visibility & Recognition, Agenda-setting, Coalition-building, and Norm Diffusion, through which participation and visibility translate into diplomatic influence. The Coalition-building also involves the building of relational capacity that can lead not just to trust or policy impact, but also to an added level of collaboration in other research, network, or policy settings, creating additional positive feedback loops.

The outer layer indicates the diplomacy-relevant outcomes that these mechanisms feed into: Legitimacy, Trust, Capacity Building, and Policy Imagination. The graphic therefore serves as an orienting map for how the subsequent evidence synthesis and outcome-focused discussion connect back to the core diplomacy mechanisms proposed here.

## Results

### Evidence synthesis: geographic, income-context, gender, career-stage, and organizational inequities

This section synthesizes current findings published since 2020 on conference equity, participation, visibility, and inclusion in public health and adjacent fields. It does not present a primary audit of conference proceedings, attendee lists, programme structures, venues, or conference-level administrative records. Instead, it integrates review-level, empirical, conceptual, policy-oriented, and practice-based sources to summarize the current evidence base on how inequities in access, recognition, and visibility shape participation in public health-related convening spaces. Source-level examples supporting the claims in this section are summarized in Supplementary Table S1.

Multiple analyses document systematic participation disparities affecting LMIC researchers and practitioners, including lower representation among speakers and leadership roles [11, 25, 26]. These geographic and income-context inequities are consequential for PHD because LMIC contexts often carry disproportionate burdens of infectious disease, humanitarian crises, and climate-related health impacts, making context-specific expertise essential for legitimate agenda-setting [25, 26]. Where conferences are dominated by Global North institutions, the risk is symbolic exclusion and a narrower menu of perceived “actionable” policy options, shaped by assumptions about system capacity, resource availability, and emergent needs [25, 35].

These geographic and income-context inequities intersect with gendered patterns of visibility, leadership, and career-stage barriers. Gender inequities in visibility and leadership are well documented across professional and governance contexts, including governing boards and high-status roles [31, 34]. Conference settings can mirror these patterns through speaker selection, session leadership, and differential recognition. In PHD, gender inequities matter not merely as representational harms but as constraints on the diversity of diplomatic networks and leadership pipelines [24, 31]. Moreover, gender inequities can interact with other dimensions, including region, caregiving expectations, institutional support, and career stage, producing compounded constraints on attendance and visibility [11, 25].

Work on “manels” provides a useful precedent for how inequities in visibility become actionable governance norms: empirical studies have quantified the persistence of all-male or male-dominated invited panels across disciplines and shown that high-status speaking roles remain unevenly distributed, prompting calls for organizers and senior invitees to adopt explicit “no-manels” rules, diversify convening teams, and treat speaker selection as an equity intervention rather than a neutral outcome [38, 39]. In roadmap terms, the same norm-to-policy pathway can be adapted to North/South equity by setting transparent criteria for plenaries, chairs, and other prestige slots so that global income and regional asymmetries are not reproduced through “who gets seen” mechanisms.

A closely related lesson concerns career stage. Youth and early-career participation can be structurally “included” yet remain weak in real influence unless design choices address gatekeeping, role power, and follow-through, an issue emphasized in recent syntheses that document a gap between participation experiences and actual policy influence, and in work explicitly framing sustainable youth engagement as moving beyond tokenism by shifting power [40, 41]. Early-career researchers and practitioners often face distinct barriers, including limited travel budgets, precarious contracts, and fewer gatekeeper connections. Access to high-visibility opportunities is often mediated by senior “sponsors” and other gatekeepers who can actively open doors by nominating early-career researchers for invited talks, awards, committees, and leadership roles, thereby shaping who becomes visible and networked in the first place [42].

In this sense, “institutional support” is not only financial or administrative; it also includes how senior leadership allocates sponsorship and gatekeeping functions that accelerate or constrain early-career trajectories. Career-stage inequity is frequently entangled with structural factors that also affect senior professionals in under-resourced settings, including limited institutional support, high clinical or public health workloads, and restricted mobility [11, 25, 26]. Interventions that target career stage without addressing geography and income context may therefore improve visibility for some, while leaving core asymmetries intact.

Beyond these structural barriers, organizational cultures and inclusion climates shape whether representation translates into meaningful influence. A broader diversity and inclusion literature suggests that representation alone does not guarantee equitable influence: inclusion climates, psychological safety, and decision-making norms shape whether diverse participants can meaningfully contribute [32, 36]. Conference formats can either enable or suppress contributions: hybrid and virtual formats can create time-zone barriers to active participation [43]; Q&A designs are known to shape who speaks up and who remains silent [22]; and English-language dominance, particularly in live Q&A, can disadvantage non-native speakers [23]. Evidence from teamwork research indicates that deep-level diversity can improve performance when inclusion is supported, but may also generate friction if not facilitated well [32, 36]. This matters for PHD because trust and consensus-building are core diplomatic functions; poorly designed inclusion can unintentionally intensify polarization or tokenism [44, 45].

Taken together, the reviewed literature suggests that conference inequity operates through more than unequal attendance. Participation gaps intersect with visibility, speaker selection, recognition practices, digital and hybrid access, language norms, costs, mobility constraints, caring responsibilities, institutional resources, and geopolitical asymmetries. Supplementary Table S1 maps these general claims to specific examples from the reviewed sources. Figure 2 synthesizes the logic that links the inequities to their diplomacy-relevant consequences. It shows how structural barriers, such as funding constraints, visa regimes, language dominance, and care responsibilities, translate into conference-level effects, including skewed participation, unequal visibility, and recurrent speaker elites, and then into systemic outcomes, such as narrow policy frames, asymmetric coalitions, and fragile legitimacy. This pathway culminates in diplomatic monocultures and motivates why the next section focuses explicitly on downstream PHD outcomes such as legitimacy, trust, coalition-building, and capacity development.

### Why inequities matter for legitimacy, trust, coalition-building, norm diffusion, and the public health diplomat pipeline

Building on the evidence synthesized above, this section explains how inequities in conference participation and visibility may shape PHD outcomes. The argument is not that conferences directly determine policy outcomes, but that they create relational, symbolic, and epistemic conditions in which legitimacy, trust, coalition-building, norm diffusion, and capacity development can emerge. Source-level examples supporting these mechanisms are summarized in Supplementary Table S2.

Legitimacy and trust in global health governance are partly produced through perceptions of fairness, representation, and responsiveness. When stakeholder groups experience global health spaces as structurally unrepresentative, trust erodes and coordination becomes harder, especially during crisis response, when compliance and shared narratives are essential [2–4]. Conference monocultures can amplify this by repeatedly privileging a narrow set of voices, which may be perceived as detached from local realities or aligned with specific geopolitical interests [25, 35].

Conferences also contribute to the pre-negotiation layer of diplomacy: they are settings where coalition-building occurs, norms diffuse, and shared frames emerge. If participation is unequal, coalition formation becomes asymmetric, and norm diffusion may reflect a limited range of institutional interests [8–10]. This is particularly consequential in the infodemic context: trusted communicators and credible networks are essential to amplifying accurate content and countering misinformation [3, 4, 37]. Where the epistemic community is narrow, the system may be less resilient against misinformation shocks and less able to tailor communication to diverse publics.

These dynamics also shape capacity building and the “public health diplomat” pipeline. Calls for strengthening PHD highlight the need for multidisciplinary training and cross-sector competencies, including systems thinking, negotiation, and communication [10, 24, 28, 29]. Conferences and multi-stakeholder fora can be training environments for these competencies, but only if access is equitable. Frameworks for health diplomacy competencies emphasize communication, facilitation, and consensus-building, which are often learned through repeated exposure to multi-actor settings [10, 24, 28]. Inequitable access thus limits not only present-day representation but also future diplomatic capacity, reinforcing monocultures over time.

In this sense, “diplomatic monocultures” should be understood not only as demographic imbalance, but as repeated patterns through which certain regions, institutions, languages, career stages, networks, and forms of expertise become more likely to define what counts as relevant knowledge in PHD. Supplementary Table S2 summarizes examples supporting this mechanism-oriented interpretation. If conferences are spaces in which recognition, networking, agenda-setting, and coalition-building take place, then inequities in access and visibility are not merely logistical or representational problems. They may also affect whose experiences and priorities become part of the diplomatic imagination of public health. This provides the rationale for the equity-by-design roadmap developed below.

### Digitalization, hybrid conferences, and the infodemic: new opportunities, new gatekeeping

Digitalization and hybrid conferencing are frequently framed as equity solutions because they reduce travel costs and can widen access [26, 33]. However, as the synthesis suggests, access alone does not guarantee recognition, visibility, or influence. Hybrid formats also introduce new gatekeeping mechanisms, including time-zone disadvantages, uneven interaction opportunities, and platform control over visibility, such as who is placed “on stage” and who remains peripheral. Supplementary Table S3 maps the intervention domains discussed in this section to examples and recommendations from the reviewed literature.

The WHO’s global strategy on digital health underscores the importance of governance and inclusive approaches in digital transformations [33]. In parallel, WHO infodemic work highlights that trust, communication infrastructures, and credible networks are pivotal during crises [4, 37]. Hybrid conferences therefore have dual significance for PHD: they can widen access, but they can also reshape who gains reputational visibility and coalition access in subtle ways. Equity-focused hybrid design requires deliberate choices about scheduling, speaker parity between in-person and remote participants, inclusive facilitation, accessible platforms, and formats that support meaningful network formation beyond physical co-presence.

Thus, digital and hybrid formats should be understood as equity mechanisms only when they are deliberately designed as such. Their value depends not merely on making attendance technically possible, but on whether remote and in-person participants have comparable opportunities to speak, interact, build networks, and be recognized. Source-level examples and recommendations supporting this equity-by-design interpretation are summarized in Supplementary Table S3.

## Discussion

### Roadmap: policy and practice options across levels

The core premise is that conference inequities are not “one-off” access problems; they are produced by interlocking design choices, resource and mobility regimes, and governance norms that shape whose expertise counts and where agendas are set. Effective reform therefore requires coordinated changes across conference organizers, institutions and funders, and global health governance actors [46-51]. Equity-by-design should be treated as a quality criterion of conferences: it shapes legitimacy, coalition-building capacity, and the diversity of policy options that can emerge from convening spaces [11, 12].

Table 1 summarizes the multi-level roadmap. It replaces a figure-based model with actionable intervention domains and concrete recommendations grounded in the reviewed literature. Level A focuses on conference organizers and professional societies, who can influence selection, programming, hybrid parity, facilitation, and visibility. Level B focuses on institutions and funders, who shape whether participation is materially possible through funding, protected time, mobility support, and recognition structures. Level C focuses on governance actors and multi-stakeholder partnerships, where conference inclusion becomes part of legitimacy infrastructure, capacity building, and agenda-setting in public health diplomacy [11, 12, 27].

### Outlook: from conference equity to resilient public health diplomacy

The synthesis suggests that conference equity is best understood as a mechanism of public health diplomacy, not a parallel fairness agenda. Conferences are part of the informal diplomatic infrastructure that shapes credibility, agenda-setting, coalition-building, and ultimately legitimacy. The policy relevance becomes clearer when we treat inequities as patterned outcomes of selection practices, mobility regimes, and convening designs that concentrate influence in a limited set of actors and settings [11, 12, 16, 17].

Two refinements follow. First, interventions that focus narrowly on early career researchers risk missing the structural core: career stage interacts with geography, income context, gender, and mobility constraints, and therefore must be embedded within intersectional equity strategies [11, 17]. Second, hybrid and virtual formats can widen access, but only if remote participation is granted parity in visibility and influence; otherwise, exclusion is simply relocated from borders and budgets to time zones, platforms, and facilitation norms [18–21]. Third, because conferences sit upstream of governance outcomes, by shaping networks and problem definitions, equity-by-design should be treated as a quality criterion of governance-adjacent convening, especially during periods of institutional turbulence when agenda-setting power is most contestable [12, 15].

### Limitations

As a narrative review, this synthesis does not provide pooled effect estimates and may be subject to selection bias toward highly visible topics and publication venues. Evidence bases vary across regions and conference types, and some mechanisms (e.g., informal coalition dynamics) are inherently difficult to quantify. Future research should combine bibliometrics with qualitative mapping of coalition formation and longitudinal tracking of leadership pipelines across regions and income contexts.

### Conclusion

Equitable participation and visibility in conferences and policy fora is a diplomacy-relevant leverage point: it shapes who becomes recognized as credible, which narratives guide agenda-setting, and which coalitions drive action. Treating conferences as informal diplomatic infrastructure clarifies why conference equity is central to PHD, particularly during governance turbulence and redistribution windows. A coordinated roadmap across organizers, institutions, funders, and governance actors can help transform diplomatic monocultures into diverse ecosystems that strengthen legitimacy, trust, and the capacity to respond to complex global health crises.
