# Supplementary material for: Diplomatic monocultures in public health diplomacy: a narrative review on conference equity, participation and visibility
Source: Public Health Rev. 2026 Jun 10;47:1609708. doi: 10.3389/phrs.2026.1609708 (PMC13291656; doi:10.3389/phrs.2026.1609708)
Supplement: Supplementary file 1 [file Supplementaryfile1.docx]

Diplomatic Monocultures in Public Health Diplomacy: A Narrative Review on Conference Equity, Participation and Visibility

**Appendix A. Descriptive thematic mapping of included sources**

To improve transparency of the narrative synthesis, we mapped all included sources to the thematic domains they informed. This mapping was conducted after the final literature set had been established and was used to clarify how the review’s conceptual structure is supported by the included literature. The mapping should not be interpreted as a systematic-review coding framework or as a basis for quantitative synthesis. Rather, it provides a transparent overview of the source types and conceptual contributions that informed the narrative review.

**Appendix Table A1**
*Thematic Mapping of Included Sources Used in the Narrative Synthesis*

| *Ref.* | *Source* | *Source type* | *Main contribution to the review* | *Thematic domain(s)* |
| --- | --- | --- | --- | --- |
| 1 | Asthana et al. (2024) | Scoping review | COVID-19 governance and public health decision-making; supports the polycrisis and governance framing. | Governance turbulence; legitimacy; crisis governance |
| 2 | Mac-Seing & Di Ruggiero (2024) | Conceptual/lessons-learned article | Equity-related lessons from COVID-19 global health governance; supports the argument that equity is central to coordinated public health action. | Governance turbulence; equity; trust |
| 3 | Joshi et al. (2025) | Summit report / field-building article | Defines public health diplomacy as a multi-actor field and supports the distinction between formal and informal diplomatic spaces. | Public health diplomacy; informal diplomacy; capacity building |
| 4 | Global Health Diplomacy Institutional Network (2025) | Competency framework | Provides competency-based framing for health diplomacy training. | Public health diplomacy; capacity building; training |
| 5 | Joshi et al. (2025) | Scoping review | Summarizes health diplomacy training, pedagogical approaches, and skills assessment. | Public health diplomacy; training; competencies |
| 6 | Velin et al. (2021) | Systematic review | Core evidence source on LMIC representation barriers at global health conferences. | Conference equity; LMIC/HIC; access barriers; visibility |
| 7 | Allotey & Reidpath (2022) | Commentary | Supports argument on diversity and representation in global health governing boards. | Governance representation; leadership; legitimacy |
| 8 | Modlin et al. (2025) | Scoping review | Synthesizes equity toolkits for international academic partnerships. | Partnership equity; LMIC/HIC; capacity building |
| 9 | Global Health 50/50 (2022) | Report | Provides evidence on power, policy, and representation in global health boards. | Leadership visibility; gender equity; governance |
| 10 | Calleja et al. (2021) | Conference/research agenda article | Supports the role of trusted communication networks in managing infodemics. | Infodemic governance; communication; trust |
| 11 | WHO (2021) | Policy report | Provides institutional framing for infodemic management during COVID-19. | Infodemic governance; crisis communication |
| 12 | Zhao et al. (2025) | Meta-review | Provides broader evidence on organizational diversity and inclusion interventions. | Inclusion climate; organizational diversity; intervention design |
| 13 | Herinek et al. (2025) | Expert position statement | Supports discussion of early-career support and professional development. | Career stage; mentorship; capacity building |
| 14 | Odeny (2021) | Conference commentary | Highlights diversity, equity, and inclusion in a global health conference context. | Conference equity; DEI; representation |
| 15 | Chikwari et al. (2024) | Empirical/qualitative or mixed policy study | Supports discussion of barriers to equitable leadership and grant funding for early- and mid-career researchers. | Career stage; LMIC/HIC; leadership; funding |
| 16 | WHO (2025) | Institutional statement | Provides current context on the United States’ announced withdrawal from WHO. | Governance turbulence; redistribution window |
| 17 | Blanchfield & Weed (2025) | Policy report | Provides background on WHO and U.S. withdrawal. | Governance turbulence; institutional reconfiguration |
| 18 | Kates et al. (2025) | Policy analysis | Supports discussion of U.S. executive actions on global health. | Governance turbulence; global health policy |
| 19 | Silburn (2025) | Conceptual/policy article | Supports systems-thinking framing and leverage-point logic. | Systems thinking; policy development; leverage points |
| 20 | Triana et al. (2021) | Meta-analysis | Supports the claim that deep-level diversity can influence team performance depending on mediating and moderating conditions. | Inclusion climate; diversity; team processes |
| 21 | WHO (2021) | Strategy document | Supports digital health governance and inclusive digital transformation framing. | Digital health; governance; inclusion |
| 22 | Wallrich et al. (2024) | Registered-report meta-analysis | Supports nuanced discussion of diversity-performance links and conditions for effective inclusion. | Inclusion climate; diversity; team performance |
| 23 | WHO (2021) | Research agenda | Supports discussion of infodemic management and communication infrastructure. | Infodemic governance; communication; trust |
| 24 | Fisher et al. (2024) | Reflection / network article | Supports practical reflections on equality, diversity, and inclusion in research and funding. | Funding equity; research networks; implementation |
| 25 | Doles et al. (2025) | Educational intervention article | Supports discussion of mentoring and leadership capacity. | Mentorship; leadership; capacity building |
| 26 | Sawhney et al. (2025) | Consensus statement | Provides a field-specific example of conference equity principles in global surgery. | Conference equity; consensus; global health practice |
| 27 | Moss et al. (2021) | Commentary / perspective | Supports discussion of post-pandemic conference models and better collaboration practices. | Hybrid/virtual conferences; access; sustainability |
| 28 | Yates et al. (2022) | Comparative conference analysis | Provides evidence on virtual events, participation, climate co-benefits, and satisfaction. | Hybrid/virtual conferences; access; sustainability |
| 29 | Doran et al. (2024) | Practical guidance article | Supports inclusive and accessible virtual/hybrid event planning. | Hybrid design; accessibility; inclusion |
| 30 | Whitfield et al. (2024) | Comparative analysis | Supports discussion of geographic equity and environmental sustainability in conference models. | Conference models; geographic equity; sustainability |
| 31 | Royal Society (2023) | Policy report | Supports discussion of visas and researcher mobility as structural barriers. | Mobility barriers; visa regimes; access |
| 32 | Gaye et al. (2024) | Position paper | Provides evidence on barriers to global engagement for African researchers. | LMIC/HIC; mobility; research equity |
| 33 | Khorsand et al. (2022) | Policy/commentary article | Supports argument that global governance gatherings can be redesigned for sustainability and equity. | Governance-adjacent convening; equity; legitimacy |
| 34 | Last et al. (2024) | Observational/commentary article | Supports discussion of gender and geographic representation at infectious diseases and clinical microbiology conferences. | Conference representation; gender; geography |
| 35 | Kumar et al. (2024) | Conceptual/policy article | Supports broader decolonizing global health framing. | Decolonization; power; global health equity |
| 36 | De Campos-Rudinsky et al. (2024) | Commentary | Supports subsidiarity and power-shifting argument in pandemic agreement/global health governance debates. | Subsidiarity; decolonization; governance legitimacy |
| 37 | Sukhera (2022) | Methodological article | Supports rationale for narrative reviews as flexible, rigorous, and practical. | Narrative review methodology |
| 38 | Louissaint et al. (2021) | Commentary / professional development article | Supports role of mentorship and sponsorship for underrepresented groups. | Mentorship; career stage; visibility |
| 39 | Ram et al. (2024) | Empirical conference attendee study | Supports discussion of how future hybrid conferences should be designed. | Hybrid conferences; participation; design |
| 40 | Jarvis et al. (2022) | Empirical conference study | Supports claim that Q&A formats affect participation and may reproduce gender gaps. | Conference interaction; gender; visibility |
| 41 | Li et al. (2025) | CHI empirical/design study | Supports discussion of non-native English speakers’ challenges in live conference Q&A. | Language dominance; accessibility; conference interaction |
| 42 | Ghanem et al. (2022) | Book chapter | Supports discussion of COVID-19, digital health, equity, and gender empowerment. | Digital health; equity; pandemic context |
| 43 | Søvold et al. (2021) | Public health article | Supports discussion of healthcare worker strain and well-being during COVID-19. | Health workforce; crisis burden; public health systems |
| 44 | Caluwaerts et al. (2023) | Multidisciplinary review | Supports caution that deliberation can interact with polarization if not well facilitated. | Deliberation; polarization; facilitation |
| 45 | Kataria & Verma (2026) | Empirical article | Supports discussion of symbolic inclusion, tokenism, and psychological safety. | Tokenism; inclusion climate; psychological safety |
| 46 | WHO (2025) | Policy document | Supports framing of well-being and governance pathways at country level. | Governance; well-being; legitimacy |
| 47 | Kamran et al. (2023) | Empirical conference study | Provides evidence on “manels” and gender representation among invited panelists. | Gender visibility; conference panels; speaker selection |
| 48 | Kibbe & Kapadia (2020) | Commentary | Supports normative argument against all-male panels and for active speaker equity. | Gender equity; conference governance |
| 49 | Ramos et al. (2026) | Scoping review | Supports discussion of youth participation and the gap between participation and actual influence. | Youth participation; tokenism; governance influence |
| 50 | Nwaozuru et al. (2025) | Editorial | Supports argument for sustainable youth engagement beyond tokenism. | Youth engagement; participation; power-sharing |
| 51 | Münter et al. (2023) | Conceptual/model article | Supports design health literacy and structured design approaches for digital development. | Design health literacy; digital health; implementation |

**Note.** The table provides a descriptive thematic mapping of the included sources rather than a formal systematic-review coding matrix. Because the article is a narrative review, sources were not coded for risk of bias or included through a reproducible screening flow. The thematic domains indicate how each source contributed to the conceptual synthesis.

**Supplementary Table S1.** *Source-level examples supporting Section 5: Evidence synthesis on conference equity, participation, visibility, and inclusion*

**Table S1a.** *Geographic and income-context inequities / LMIC-HIC participation gaps*

| Manuscript claim supported | Source | Evidence type | Specific source-level finding/example | Relevance to Section 5 |
| --- | --- | --- | --- | --- |
| LMIC researchers and practitioners are systematically underrepresented in global health conferences. | Velin et al. [6] | Systematic review of global health conference equity literature | The review included 46 articles describing 112 conferences with 254,601 attendees. Only 4% of conferences were hosted in low-income countries. Among attendees for whom affiliation was disclosed, 39% were from LMICs. | Directly supports the claim that global health conference participation remains structurally skewed toward HIC actors. |
| Conference inequity concerns access, active engagement, and influence, not attendance alone. | Velin et al. [6] | Conceptual definition within systematic review | The authors define “conference equity” as equitable active engagement, influence, and access regardless of country of origin, location, available funds, or affiliation, through mitigation of barriers and enhancement of facilitators. | Supports the manuscript’s distinction between being present at a conference and being able to meaningfully participate or influence agendas. |
| LMIC attendees face systemic barriers to conference participation. | Velin et al. [6] | Systematic review | LMIC attendance was limited by high travel costs, visa restrictions, and lower acceptance rates for research presentations. The authors recommend visa-friendly locations, travel scholarships, and mentorship programmes. | Supports the claim that participation gaps are produced by financial, administrative, and gatekeeping mechanisms rather than individual choice alone. |
| Global health conferences are often geographically concentrated in HIC settings. | Velin et al. [6] | Systematic review | The majority of reviewed conferences were hosted in HICs, with conference geography reflecting wider global health power asymmetries. | Supports the claim that conference location is part of the infrastructure through which Global North dominance is reproduced. |
| Visa regimes create unequal mobility barriers. | Velin et al. [6] | Systematic review | The review reports that visa barriers disproportionately affect LMIC participants and discusses evidence that academics from Africa and Asia report substantially more visa challenges than academics from Europe or North America. | Supports the manuscript’s statement that visa regimes are not neutral logistical issues, but structural access barriers. |
| LMIC underrepresentation has consequences beyond attendance. | Velin et al. [6] | Systematic review conclusion | The authors argue that conference inequity creates missed opportunities for decision-making, networking, advocacy, capacity building, professional development, and reciprocal learning between LMIC and HIC stakeholders. | Supports the manuscript’s argument that conference inequity matters for public health diplomacy because it affects relational and epistemic infrastructure. |
| Global surgery conferences underrepresent LMIC frontline providers. | Sawhney et al. [26] | Consensus statement based on literature review and modified Delphi process | The consensus statement identifies financial barriers, geopolitical barriers, and accessibility barriers to LMIC participation, including registration and travel costs, visa issues, distance, time zones, language barriers, lack of mentorship, and authorship inequity. | Provides a conference-specific example from global surgery showing that the mechanisms identified by Velin et al. also appear in a clinical global health field. |
| Practical conference reforms can directly target LMIC participation barriers. | Sawhney et al. [26] | Consensus recommendations | Recommended actions include fee waivers, travel grants, hybrid conferences, visa support, translation services, mentorship opportunities, targeted advertising in LMIC forums, and relocating conferences to LMICs. | Supports the manuscript’s equity-by-design framing: conference inequity can be addressed through intentional governance and design decisions. |
| Virtual models can substantially broaden geographic participation. | Whitfield et al. [30] | Comparative analysis of in-person, virtual, and hybrid TB conferences | A virtual TB conference hosted 2.5 times more participants than the average in-person conference and included participants from more than twice as many countries. It attracted 4.5 times more participants from high-TB-burden countries. | Supports the claim that virtual participation can widen access for participants from high-burden and geographically underrepresented settings. |
| Virtual access can broaden participation without clear loss of perceived scientific quality. | Whitfield et al. [30] | Comparative conference model analysis | Virtual conference scientific quality ratings were comparable to ratings for in-person conferences. The virtual model also dramatically reduced estimated carbon emissions. | Supports the claim that access-oriented digital models need not undermine perceived conference quality. |
| Hybrid formats may still reproduce HIC dominance in the physical conference space. | Whitfield et al. [30] | Comparative conference model analysis | In the hybrid TB conference, 91% of in-person participants were from HICs, whereas virtual participation was more geographically diverse. | Supports the manuscript’s caution that hybrid access may widen attendance while leaving in-person visibility and networking concentrated among HIC participants. |
| Global health partnerships reproduce inequities in recognition and outputs, including conference presentation. | Modlin et al. [8] | Scoping review of equity toolkits for international academic partnerships | The review identifies asymmetries in power, finances, resources, academic recognition, authorship, conference presentation, data ownership, and intellectual property as recurring concerns in international academic partnerships. | Supports the manuscript’s link between conference visibility and wider partnership inequities in global health. |
| Equity tools identify domains relevant to participation, visibility, governance, and capacity. | Modlin et al. [8] | Scoping review | Seventeen equity toolkits were identified. Common domains included oversight, partnership dynamics, ethical foundations, contextual factors, procedures and activities, and capacity. Recurrent themes included stakeholder engagement, communication, outputs, roles, funding, and data/product management. | Supports the claim that equity must be designed across governance, process, output, and capacity domains rather than reduced to attendance. |
| LMIC research leadership is constrained by funding and institutional asymmetries. | Chikwari et al. [15] | Workshop-based correspondence / thematic analysis | The authors identify lack of individual and institutional support and flawed funding structures as barriers for early- and mid-career researchers in LMIC settings. | Supports the claim that conference participation inequities are connected to broader leadership and research-capacity asymmetries. |

**Table S1b.** *Gender inequities, leadership visibility, and prestige roles*

| *Manuscript claim supported* | *Source* | *Evidence type* | *Specific source-level finding/example* | *Relevance to Section 5* |
| --- | --- | --- | --- | --- |
| Gender and geographic inequities persist in global health governance leadership. | Global Health 50/50 [9] | Governance report / board composition analysis | The report reviewed board composition in organizations active in global health and showed strong imbalances by gender, nationality, and income context. Board representation was particularly limited for women from low-income countries. | Supports the claim that gender inequities in conference visibility mirror broader leadership inequalities in global health governance. |
| Global health boards remain dominated by HIC nationals. | Global Health 50/50 [9] | Governance report | The report found that a large majority of board seats were held by HIC nationals, with particularly low representation of LIC nationals and women from LICs. | Supports the manuscript’s intersectional framing of gender, geography, and income-context inequity. |
| Board diversity is not only symbolic; it shapes whose perspectives guide global health organizations. | Allotey & Reidpath [7] | Commentary on Global Health 50/50 report | The authors argue that diversity in global health governing boards matters because boards shape policy, priorities, and organizational legitimacy. | Supports the manuscript’s claim that visibility and leadership representation affect governance authority, not only fairness. |
| Meeting formats can enable or restrict participation by LMIC-based actors. | Allotey & Reidpath [7] | Commentary | The authors emphasize that organizations need to consider where, when, and how meetings are held and whether meeting formats enable participation by people based in LMICs. | Supports the claim that inclusion requires design choices that allow meaningful participation. |
| Gender and geography remain uneven in high-prestige conference roles. | Last et al. [34] | Retrospective observational analysis of infectious disease and clinical microbiology conferences | The study examined gender and geographic representation among chairs and speakers, including high-prestige sessions. Women were less represented in prestigious keynote lecture roles than in overall speaker/chair roles. | Supports the claim that overall participation statistics can obscure inequities in high-status visibility slots. |
| LMIC representation remains low in high-visibility infectious disease conference roles. | Last et al. [34] | Conference programme analysis | The study found that LMIC representation among speakers and chairs remained low and was particularly limited in prestigious keynote speaker positions. | Provides a direct conference-specific example supporting the claim that geography and income context shape visibility. |
| Programme committee action can improve representation. | Last et al. [34] | Conference analysis / commentary | The authors describe how explicit committee-level gender-equity efforts can improve representation in prestigious speaking positions. | Supports the manuscript’s claim that speaker selection is an actionable governance mechanism. |
| All-male or male-dominated invited panels remain a measurable form of visibility inequity. | Kamran et al. [47] | Retrospective analysis of invited panels at ASCO Annual Meetings, 2018–2021 | Across 670 sessions, 81 were manels. Among 2,475 panelists, 47.7% were women. Manels decreased over time from 17.4% in 2018 to 9.9% in 2021. | Directly supports the manuscript’s use of “manels” as an example of measurable visibility inequity. |
| Gender representation can improve over time but remain uneven by topic and session type. | Kamran et al. [47] | Retrospective conference analysis | Women’s panel representation increased over time, but women remained underrepresented in some topic areas, including genitourinary cancers and translational/preclinical sciences. | Supports the claim that equity gains may be partial and uneven across prestige domains and thematic fields. |
| Invitations to speak at major meetings affect career advancement. | Kamran et al. [47] | Conference analysis with discussion of academic advancement | The authors state that invitations to speak at major meetings provide visibility and are a metric used by promotion and tenure committees. | Supports the manuscript’s argument that speaker selection is not merely symbolic, but affects professional authority and leadership pipelines. |
| Manels can be reduced through deliberate awareness and programme committee instructions. | Kamran et al. [47] | Discussion drawing on prior professional-society experience | The paper describes evidence that presenting representation data to programme committees and instructing committees to avoid all-men sessions increased women speaker representation and reduced manels. | Supports the claim that speaker diversity can be governed through explicit selection norms. |
| Manels reduce diversity of perspectives and harm professional recognition. | Kibbe & Kapadia [48] | Invited commentary | The authors argue that manels limit perspectives, send exclusionary messages to junior colleagues, reduce women’s recognition and promotion opportunities, and undermine the quality of academic panels. | Supports the manuscript’s claim that visibility inequities affect knowledge diversity and career pathways. |
| Avoiding manels is a shared responsibility of organizers and invited speakers. | Kibbe & Kapadia [48] | Invited commentary | The authors recommend that invitees speak up, suggest alternative panelists, decline non-diverse panels when appropriate, and use pledges or public accountability mechanisms. | Supports the manuscript’s framing of speaker selection as an equity intervention rather than a neutral outcome. |
| Gendered visibility gaps occur not only in formal panels, but also in Q&A interactions. | Jarvis et al. [40] | Two-study empirical analysis of Q&A participation at academic conferences | Men disproportionately participated in live Q&A sessions, while women reported lower comfort, greater fear of backlash, and more anxiety-related withholding of questions. | Supports the claim that conference formats shape who speaks and whose contributions become visible. |
| Q&A participation signals authority and belonging. | Jarvis et al. [40] | Empirical paper / theoretical framing | The authors describe Q&A as a highly visible interactional space because limited time means only a few people can ask questions; those who do so signal recognition, authority, and belonging. | Supports the manuscript’s claim that visibility includes interactional opportunities, not only formal speaking roles. |

**Table S1c.** *Career-stage barriers, sponsorship, mentorship, and gatekeeping*

| *Manuscript claim supported* | *Source* | *Evidence type* | *Specific source-level finding/example* | *Relevance to Section 5* |
| --- | --- | --- | --- | --- |
| Early- and mid-career researchers in LMIC settings face structural barriers to leadership. | Chikwari et al. [15] | Workshop-based thematic analysis | The authors identify lack of individual support, weak institutional support, and flawed funding structures as major barriers for EMCRs in LMIC settings. | Supports the manuscript’s claim that career-stage inequity intersects with geography and income context. |
| LMIC leadership requires locally defined research priorities and funding access. | Chikwari et al. [15] | Global health partnership analysis | The paper argues that equitable leadership requires LMIC researchers to secure grant funding and lead research based on locally defined priorities. | Supports the manuscript’s argument that inequitable participation narrows agenda-setting and perceived policy options. |
| Mentorship is a key mechanism for career development and leadership. | Chikwari et al. [15] | Workshop-based thematic analysis | Participants identified lack of mentorship support as a key gap for EMCRs transitioning toward independence and future leadership. | Supports the claim that institutional support includes relational and developmental infrastructure. |
| Weak networks limit career progression and grant competitiveness. | Chikwari et al. [15] | Workshop-based thematic analysis | EMCRs identified strong networks as important for grant writing, research ideas, multidisciplinary teams, and funder confidence. | Supports the manuscript’s claim that gatekeeper connections and network access shape who becomes visible and competitive. |
| Reform requires institutional support, mentorship, dedicated funding, and South-South collaboration. | Chikwari et al. [15] | Recommendations | The authors recommend investment in LMIC research institutions, mentorship and networking systems, dedicated EMCR/LMIC funding streams, long-term relationships with decision-makers, and South-South collaborations. | Supports the manuscript’s broader claim that career-stage equity requires structural support, not only individual capacity building. |
| Early-career researchers require formal support pathways. | Herinek et al. [13] | Expert position statement | The position statement identifies needs including distinct career paths, robust funding strategies, nurturing and diverse intellectual cultures, connection to practice, and investment in leadership, advocacy, and coaching. | Supports the claim that early-career visibility depends on institutionalized support structures. |
| Conference attendance and presentation are part of early-career development. | Herinek et al. [13] | Expert position statement | Recommended implementation steps include connecting ECRs to professional and research networks and supporting them to attend and present at local, national, and international conferences. | Directly supports the manuscript’s framing of conferences as part of the career-stage pipeline. |
| Mentorship must be linked to sponsorship to create high-visibility opportunities. | Louissaint et al. [38] | Commentary / action-oriented guidance | The authors distinguish mentorship from sponsorship and argue that sponsors help early-career scholars access opportunities such as visibility, promotion, and leadership pathways. | Supports the manuscript’s claim that senior gatekeepers shape who becomes visible and networked. |
| Underrepresented early-career scholars benefit from formal mentorship and professional-society involvement. | Louissaint et al. [38] | Guidance article | Recommended actions include formal mentorship programmes, early professional-society involvement, normalization of sponsorship, and valuing diversity-related work in promotion. | Supports the claim that institutional support is not only financial, but also relational and reputational. |
| Youth participation can be formally included but weak in actual influence. | Ramos et al. [49] | Scoping review of youth participation in local governance | The review finds that youth participation is widely endorsed but that outcomes depend on institutional design, gatekeeping dynamics, and the conditions under which participation occurs. | Supports the manuscript’s claim that being “included” does not guarantee real influence. |
| Youth participation should not be treated as inherently democratic or effective. | Ramos et al. [49] | Scoping review | The authors argue for closer examination of design logics and the conditional nature of positive participation outcomes. | Supports the manuscript’s caution against tokenistic inclusion and reinforces the need for equity-by-design. |
| Sustainable youth engagement requires power-sharing rather than tokenistic involvement. | Nwaozuru et al. [50] | Editorial / implementation-oriented synthesis | The editorial frames meaningful youth engagement as moving beyond tokenism by placing young people at the center of programme design, implementation, evaluation, and dissemination. | Supports the manuscript’s claim that participation must include influence, role power, and follow-through. |
| Youth engagement should involve participation across the whole implementation process. | Nwaozuru et al. [50] | Editorial | The article highlights youth involvement in research groups, innovation bootcamps, youth participatory action research, design, delivery, evaluation, analysis, implementation, and dissemination. | Supports the manuscript’s distinction between symbolic inclusion and sustained engagement. |

**Table S1d.** *Organizational cultures, inclusion climates, psychological safety, and tokenism*

| *Manuscript claim supported* | *Source* | *Evidence type* | *Specific source-level finding/example* | *Relevance to Section 5* |
| --- | --- | --- | --- | --- |
| Representation alone does not guarantee inclusion or influence. | Zhao et al. [12] | Systematic meta-review of organizational D&I interventions | The meta-review identified 12 categories of D&I interventions across 37 reviews and emphasized that evidence varies substantially by intervention type, diversity dimension, and outcome. | Supports the manuscript’s claim that equity requires specific organizational practices, not mere representational diversity. |
| D&I interventions often have limited or mixed effects unless goals and mechanisms are clear. | Zhao et al. [12] | Meta-review | Diversity training showed comparatively higher-quality evidence, but effects were mainly limited to awareness and learning outcomes. Evidence for recruitment, leave, and compensation policies was mixed. | Supports cautious wording: interventions should not be assumed effective without attention to design, implementation, and outcomes. |
| Organizations often invest in D&I without clear outcome expectations. | Zhao et al. [12] | Meta-review / research-practice gap analysis | The authors highlight that organizations may implement D&I initiatives without clear goals or knowledge of likely positive or negative outcomes. | Supports the manuscript’s argument for equity-by-design as a governance and quality criterion. |
| Deep-level diversity can create both potential benefits and process challenges. | Triana et al. [20] | Meta-analysis of 94 papers, 280 effect sizes, 24,425 teams | Deep-level diversity was associated with fewer positive emergent states, fewer positive team processes, and more team conflict. | Supports the claim that diversity must be supported by inclusive processes to become productive. |
| Diversity affects team functioning through mediating processes. | Triana et al. [20] | Meta-analysis | The relationship between deep-level diversity and performance operated indirectly through positive emergent states, positive team processes, and conflict. | Supports the manuscript’s claim that inclusion climates and facilitation matter for whether diversity improves or undermines collaboration. |
| Positive team processes include communication, collaboration, cooperation, and information sharing. | Triana et al. [20] | Meta-analysis / conceptual model | The paper defines positive team processes as collaboration, communication, cooperation, information sharing, and helping. | Supports the manuscript’s statement that conference facilitation and discussion formats shape whether diverse participants can contribute meaningfully. |
| Team diversity does not automatically translate into performance benefits. | Wallrich et al. [22] | Registered report meta-analysis of 615 reports and 2,638 effect sizes | Average linear relationships between demographic, job-related, and cognitive diversity and team performance were statistically significant but very small. | Supports a nuanced claim: diversity is valuable, but its effects depend on context, task, and facilitation. |
| Context conditions whether diversity becomes productive. | Wallrich et al. [22] | Registered report meta-analysis | Diversity-performance associations were more positive for complex tasks or tasks requiring creativity and innovation, and in contexts lower in collectivism and power distance. | Supports the claim that conference inclusion requires conditions enabling participants to use diverse perspectives productively. |
| Diversity may provide cognitive resources but also trigger conflict or communication barriers. | Wallrich et al. [22] | Meta-analysis / theoretical framing | The paper contrasts cognitive-resource benefits of diversity with social-identity pathways involving conflict, communication barriers, and anxiety. | Supports the manuscript’s statement that poorly facilitated inclusion can generate friction rather than meaningful consensus-building. |
| Symbolic inclusion and tokenism can undermine psychological safety. | Kataria & Verma [45] | Cross-sectional organizational study | Perceived tokenism was negatively associated with psychological safety and contextual performance, while psychological safety was positively associated with contextual performance. | Supports the manuscript’s claim that symbolic inclusion can undermine meaningful participation. |
| Less experienced professionals may experience stronger tokenism-related disadvantages. | Kataria & Verma [45] | Cross-sectional study | The study reports that less experienced professionals showed higher perceived tokenism and lower contextual performance than more experienced professionals. | Supports the intersection between career-stage vulnerability and inclusion climate. |
| D&I strategies should move beyond numerical representation. | Kataria & Verma [45] | Organizational study | The authors argue that inclusion strategies should go beyond numerical representation and address communication, burnout, and inclusive leadership. | Supports the manuscript’s claim that participation must be accompanied by psychological safety and meaningful voice. |
| Deliberative interaction can reduce or increase polarization depending on design. | Caluwaerts et al. [44] | Multidisciplinary systematic review | The review finds depolarizing effects when discussions follow deliberative-democracy principles, but polarizing effects when they do not; outcomes depend on design factors. | Supports the manuscript’s cautious statement that poorly designed inclusion can intensify polarization or tokenism. |
| Consensus-building requires structured interaction. | Caluwaerts et al. [44] | Multidisciplinary review | The authors emphasize that communication effects depend on whether the interaction is designed according to deliberative principles. | Supports the claim that conference formats require facilitation quality to support trust and consensus-building. |

**Table S1e.** *Hybrid/virtual formats, Q&A, language barriers, and participation quality*

| *Manuscript claim supported* | *Source* | *Evidence type* | *Specific source-level finding/example* | *Relevance to Section 5* |
| --- | --- | --- | --- | --- |
| Many attendees prefer hybrid conference models. | Ram et al. [39] | Survey and directed content analysis of conference attendees | Among 813 respondents expressing a preference, 56.9% preferred hybrid conference formats, 32.0% preferred in-person formats, and 11.1% preferred virtual formats. | Supports the claim that hybrid conferences respond to attendee preferences and may be an important access model. |
| Hybrid design requires deliberate choices about timing, interaction, and programme distribution. | Ram et al. [39] | Survey | Among respondents considering a hybrid meeting, 67.4% preferred virtual sessions both during the in-person conference and spread throughout the year. | Supports the manuscript’s claim that hybrid access should be designed rather than treated as passive streaming. |
| Virtual and hybrid formats can widen access but may weaken interaction. | Ram et al. [39] | Survey and content analysis | Respondents valued accessibility and flexibility, but also identified challenges around interaction, networking, digital fatigue, technology, and time zones. | Supports the balanced claim that hybrid formats can enable and suppress participation. |
| Conference attendees ask for better interaction opportunities and attention to time zones/languages. | Ram et al. [39] | Survey-based attendee recommendations | Suggested improvements included better interaction, technical reliability, fewer simultaneous sessions, longer access to recordings, and consideration of time zones and languages. | Provides concrete support for the manuscript’s statement that time-zone and language design affect active participation. |
| Virtual and hybrid events can reproduce exclusion if accessibility is not planned. | Doran et al. [29] | Review article / practical guidance | The authors state that virtual and hybrid events can create opportunities for groups previously excluded from in-person formats, but without careful inclusion and accessibility planning they can replicate the biases and exclusions of in-person events. | Supports the manuscript’s claim that digital formats are not automatically equitable. |
| Accessibility needs to be planned across the full event lifecycle. | Doran et al. [29] | Review article / guidance | The article organizes recommendations around pre-event planning, during-event delivery, and post-event practices. | Supports the manuscript’s lifecycle approach to conference equity and hybrid design. |
| Digital accessibility can enable more meaningful engagement when deliberately designed. | Doran et al. [29] | Review article | The authors argue that inclusive and accessible design can help participants engage more meaningfully and include contributions from groups with limited access to in-person events. | Supports the manuscript’s equity-by-design framing for hybrid and virtual events. |
| Live Q&A is a language-intensive and high-pressure conference format. | Li et al. [41] | Interview study with non-native English-speaking academics | Live Q&A at English-based international conferences requires real-time comprehension and response in a non-native language under stress. The format is spontaneous, time-sensitive, and difficult to prepare for. | Directly supports the claim that English-language dominance can suppress participation in live Q&A. |
| Non-native English-speaking presenters face dual cognitive demands during Q&A. | Li et al. [41] | Interview study | Presenters must simultaneously manage research content and language barriers, which can hinder confident and full participation in scientific discussion. | Supports the claim that language norms shape who can speak and be recognized in conference settings. |
| Language-support tools may help, but are inconsistently implemented. | Li et al. [41] | Interview study / design implications | The study discusses real-time translation, transcription, captioning, and written-question platforms, but notes that meaningful linguistic support remains rare or inconsistently available at conferences. | Supports the manuscript’s claim that inclusive conference design requires formal language accommodations. |
| Q&A participation is gendered and affects visibility. | Jarvis et al. [40] | Empirical study of Q&A interactions and attendee attitudes | Men disproportionately participated in Q&A sessions, while women were less comfortable participating and more likely to fear backlash or hold back questions because of anxiety. | Supports the claim that conference formats shape who speaks up and who remains silent. |
| Q&A is a visible signal of recognition and authority. | Jarvis et al. [40] | Empirical study / theoretical framing | The authors describe Q&A participation as highly visible because only a few attendees can ask questions, making question-asking a signal of authority and belonging. | Supports the manuscript’s argument that visibility extends beyond formal speaking roles. |
| Virtual conference models can increase geographic reach and reduce environmental burden. | Whitfield et al. [30] | Comparative analysis | The virtual TB conference reached more countries and more high-TB-burden countries than in-person conferences, while producing far lower estimated carbon emissions. | Supports the claim that digital formats can advance access and sustainability. |
| Hybrid models can widen attendance but may leave prestige and networking concentrated in the physical venue. | Whitfield et al. [30] | Comparative analysis | In the hybrid conference, in-person attendance remained heavily HIC-dominated, whereas virtual participation broadened geographic inclusion. | Supports the manuscript’s caution that hybrid access must address visibility and influence, not merely attendance. |

**Supplementary Table S2.** *Source-level examples supporting Section 6: Why inequities matter for public health diplomacy outcomes*

**Table S2a.** *Legitimacy and trust in global health governance*

| *Manuscript claim supported* | *Source* | *Evidence type* | *Specific source-level finding/example* | *Relevance to Section 6* |
| --- | --- | --- | --- | --- |
| Legitimacy and trust in global health governance depend on fairness, representation, responsiveness, accountability, and equity. | Asthana et al. [1] | Scoping review of governance and decision-making during COVID-19 | The review defines governance as decision-making and implementation, and identifies accountability, transparency, equity, participation, and rule of law as elements of good governance needed for cohesive pandemic response. | Directly supports the claim that legitimacy in crisis governance depends on perceived fairness, representation, and accountable decision-making. |
| COVID-19 exposed weaknesses in global health governance and equity-oriented decision-making. | Mac-Seing & Di Ruggiero [2] | Policy brief combining qualitative study and scoping review | The authors report that global health governance and national governments failed to sufficiently promote global health solidarity and ensure equitable health outcomes during rapid COVID-19 decision-making. | Supports the claim that governance spaces perceived as inequitable can undermine trust and coordination. |
| Limited coordination among governance actors contributed to fragmented COVID-19 responses. | Mac-Seing & Di Ruggiero [2] | Policy brief / qualitative and scoping-review synthesis | The authors state that nationalism and limited coordination among governance actors contributed to fragmented COVID-19 public health responses. | Supports Section 6’s statement that trust and coordination become harder when governance spaces are fragmented or unrepresentative. |
| Equity needs to be linked to funding and accountability. | Mac-Seing & Di Ruggiero [2] | Policy brief | The authors emphasize the importance of linking equity considerations to funding and accountability in COVID-19 responses. | Supports the mechanism that equity is not merely symbolic but must be embedded in governance resources and accountability structures. |
| Vulnerable and marginalized groups were disproportionately affected by negative consequences of governance processes and public health measures. | Mac-Seing & Di Ruggiero [2] | Policy brief | The authors report that when COVID-19-related consequences were negative, they affected groups living and working in conditions of vulnerability and marginalization more strongly. | Supports the claim that unrepresentative governance can produce or amplify inequitable outcomes. |
| Global health governance requires contextually appropriate, locally responsive decision-making. | De Campos-Rudinsky et al. [36] | Viewpoint on subsidiarity and pandemic governance | The authors argue that subsidiarity would empower local units to make decisions at the level closest to the issue, fostering collaboration, coordination, cooperation, and contextually appropriate responses. | Supports the claim that legitimacy depends on responsiveness to local realities and not only centralized authority. |
| Global solidarity requires balancing local agency with non-abandonment. | De Campos-Rudinsky et al. [36] | Viewpoint | The paper defines subsidiarity through agency and non-abandonment; it cautions that agency without collaboration can become nationalism, while non-abandonment without agency can become patronizing assistance. | Supports the manuscript’s concern that global health spaces need inclusive, non-paternalistic forms of participation. |
| Power asymmetries and knowledge hierarchies undermine decolonial and equitable global health governance. | Kumar et al. [35] | Review / conceptual analysis | The authors argue that global health research remains shaped by power asymmetries, knowledge hierarchies, skewed resource distribution, and limited commitments to capacity strengthening. | Supports the manuscript’s claim that narrow epistemic communities can shape what counts as relevant knowledge in PHD. |
| Existing equity checklists may not sufficiently shift decision-making power. | Kumar et al. [35] | Review / conceptual analysis | The authors argue that guidelines and checklists for equitable research partnerships often do not adequately address underlying power asymmetries and biases, calling instead for redistributing decision-making power and resources. | Supports Section 6’s broader argument that inclusion must affect authority and agenda-setting, not just formal presence. |
| Global governance gatherings themselves can affect legitimacy and inclusion. | Khorsand et al. [33] | Commentary on World Health Assemblies | The authors describe World Health Assemblies as central global health decision-making spaces and argue that virtual formats enabled broader access to diverse voices while reducing cost and visa barriers. | Supports the conference-to-governance bridge: access to convening spaces affects who is represented in policy-adjacent discussions. |
| Returning to pre-pandemic in-person formats can reintroduce exclusion. | Khorsand et al. [33] | Commentary | The authors warn that resuming pre-pandemic travel patterns during unequal vaccine access could further exclude already underrepresented populations. | Supports the claim that convening design affects perceived fairness and legitimacy of global health governance. |

**Table S2b.** *Coalition-building, norm diffusion, and the pre-negotiation layer of diplomacy*

| *Manuscript claim supported* | *Source* | *Evidence type* | *Specific source-level finding/example* | *Relevance to Section 6* |
| --- | --- | --- | --- | --- |
| Public health diplomacy depends on cross-disciplinary collaboration, communication, negotiation, and consensus-building. | Joshi et al. [3] | Policy brief summarizing Public Health Diplomacy Summit | The summit developed a working definition of PHD emphasizing cross-disciplinary collaborations, communication, negotiation, and consensus-building. | Directly supports the claim that PHD operates through relational and communicative mechanisms. |
| Public health diplomacy requires collaboration across governments, multilateral organizations, NGOs, academia, and the private sector. | Joshi et al. [3] | Policy brief | The paper states that PHD fosters collaboration across governments, multilateral organizations, NGOs, academia, and the private sector to advance global health priorities and shared development/security goals. | Supports the claim that conferences and summits can function as multi-actor coalition-building spaces. |
| The pandemic showed the need to combine formal and informal diplomacy. | Joshi et al. [3] | Policy brief | The authors state that pandemic lessons stress the importance of combining formal diplomacy with informal diplomacy involving non-state actors such as NGOs and private enterprises. | Supports your framing of conferences as informal diplomatic infrastructure around formal diplomacy. |
| PHD summit formats can generate action plans and institutionalize diplomacy capacity. | Joshi et al. [3] | Summit report / policy brief | The summit produced a 9-point action plan to establish a global framework, launch capacity-building initiatives, and institutionalize PHD as a public health discipline. | Supports the claim that convening spaces can shape shared frames and future institutional pathways. |
| Health diplomacy requires coalition-building and advocacy as core competencies. | Global Health Diplomacy Institutional Network [4] | Competency framework | The framework identifies five key competencies, including coalition-building and advocacy, defined as the ability to build trust, networks, and alliances across sectors and influence decision-making. | Directly supports the claim that coalition-building is not incidental but central to health diplomacy. |
| Cross-cultural and interpersonal communication is a core diplomacy competency. | Global Health Diplomacy Institutional Network [4] | Competency framework | The framework lists cross-cultural and interpersonal communication as a key competency, requiring collaboration across cultural and political contexts and adaptation of communication strategies to different audiences. | Supports the argument that unequal access to communication spaces can affect diplomatic capacity. |
| Public health diplomacy requires political analysis and the ability to influence agendas and priorities. | Global Health Diplomacy Institutional Network [4] | Competency framework | The framework defines political analysis and diplomacy skills as the ability to navigate political dynamics and decision-making structures to influence health agendas and priorities. | Supports the claim that agenda-setting is part of the diplomacy mechanism. |
| Health diplomacy training targets negotiation, governance, cross-sector collaboration, and crisis management. | Joshi et al. [5] | Scoping review of health diplomacy training | The review found that curricula and training initiatives commonly addressed negotiation, governance, international policy, cross-cultural communication, leadership, and crisis management. | Supports the claim that PHD competencies are learned through exposure to multi-actor and negotiation-oriented settings. |
| Training initiatives often use simulations, workshops, peer learning, and problem-based approaches. | Joshi et al. [5] | Scoping review | Included initiatives ranged from simulations and workshops to semester-long curricula and flexible competency frameworks; common pedagogies included simulation-based experiential learning, peer-to-peer learning, problem-based approaches, and competency-based approaches. | Supports the manuscript’s claim that multi-stakeholder fora and repeated exposure can be training environments for diplomacy skills. |
| Public health policy benefits from systems approaches because problems involve interdependent stakeholders. | Silburn [19] | Opinion/review on systems thinking in policy development | Systems thinking is described as a framework for understanding interconnections among public health systems and anticipating policy consequences; it helps improve stakeholder coordination and adaptive responses. | Supports the claim that PHD outcomes depend on relational and systemic coordination rather than isolated technical expertise. |
| Systems thinking explicitly highlights stakeholder interconnections across governmental, non-governmental, and public actors. | Silburn [19] | Systems thinking article | The paper states that systems thinking identifies interconnections between complex and loosely coupled systems of stakeholders, including government, NGOs, and the general population. | Supports the conceptual bridge between conferences as relational spaces and governance outcomes. |

**Table S2c.** *Infodemic governance, trusted communicators, and credible networks*

| *Manuscript claim supported* | *Source* | *Evidence type* | *Specific source-level finding/example* | *Relevance to Section 6* |
| --- | --- | --- | --- | --- |
| Infodemics can erode trust in health authorities and public health responses. | Calleja et al. [10] | Original paper / WHO Infodemiology Conference research agenda | The paper defines an infodemic as an overflow of variable-quality information during a public health event that can cause confusion, risk-taking, harmful behaviours, and erosion of trust in health authorities and responses. | Directly supports the claim that trust and credible information networks are central during crisis response. |
| Infodemic management requires multidisciplinary and cross-sector collaboration. | Calleja et al. [10] | WHO conference-based research agenda | The first WHO Infodemiology Conference included 110 participants from over 35 countries and diverse disciplines, using structured expert judgment to develop a research agenda. | Supports the claim that conference-like expert convenings can form shared frames and research priorities. |
| Infodemic response requires partnerships beyond usual public health networks. | Calleja et al. [10] | Original paper | The authors state that health authorities needed to build partnerships with fact-checkers, media and journalists, social media/search/digital platforms, community organizations, civil society, and others. | Supports the claim that narrow epistemic communities are insufficient for misinformation resilience. |
| Infodemic response is a whole-information-ecosystem challenge. | Calleja et al. [10] | Original paper | The paper argues that COVID-19 infodemic management cannot rely on communication and messaging alone; it must account for the information ecosystem and interventions across individual, community, medium, platform, and policy levels. | Supports your claim that credible networks and diverse communicators are necessary to tailor communication to diverse publics. |
| Trust is a key element in resilient communities. | Calleja et al. [10] | Conference research agenda | Conference participants emphasized the interplay between information ecosystem actors and community resilience, identifying trust as key to building resilient communities and establishing trustworthy information sources. | Directly supports the Section 6 sentence linking trusted communicators and credible networks to misinformation resilience. |
| Communities need easy and equitable access to trustworthy information sources. | Calleja et al. [10] | Conference research agenda | Participants emphasized the need to identify trustworthy information sources and ensure easy and equal access to them. | Supports the claim that inequitable information ecosystems can weaken public health response capacity. |
| Infodemic management aims to deliver the right information at the right time and in the right format. | WHO [11] | WHO overview report | The report states that infodemic management aims to ensure that people have the right information, at the right time, in the right format, so they can adopt protective behaviours. | Supports the claim that communication infrastructures are central to crisis coordination. |
| WHO’s infodemic work explicitly includes social listening, risk communication, resilience to misinformation, community empowerment, and global conferences. | WHO [11] | WHO overview report | The report’s structure includes sections on social listening, risk communication, misinformation resilience, community empowerment, high-level events/global conferences, and regional/country approaches. | Supports the manuscript’s claim that infodemic governance depends on networks, trust, and credible communication infrastructure. |
| Infodemic management requires online and offline interventions adapted to local communities. | WHO [11] | WHO overview report | The report emphasizes that misinformation/disinformation harms require interventions online and offline and that people in every country and community need support to manage infodemics. | Supports your argument that narrow epistemic communities are less able to tailor communication to diverse publics. |
| WHO’s infodemic research agenda resulted from an online global conference and prioritization process. | WHO [23] | WHO public health research agenda | The research agenda was the main outcome of the first WHO global infodemiology conference, held online between June and July 2020, followed by prioritization exercises through October 2020. | Supports the claim that convening spaces can structure research priorities and shared response agendas. |
| Infodemic work requires multiple disciplines and a global community of experts. | WHO [23] | WHO research agenda | WHO acknowledges experts from around the world as participants, including public health, communication, journalism, digital technology, social behaviour, marketing, ethics, and related fields. | Supports the claim that epistemic diversity is necessary for credible infodemic governance. |
| Global health conferences can foreground vaccine equity, misinformation, and pandemic governance. | Odeny [14] | Perspective on CUGH 2021 conference | Odeny describes CUGH 2021 as a virtual conference with more than 5,000 delegates across disciplines, with major agenda items including COVID-19, vaccine equity, and the vaccine infodemic. | Provides a concrete conference example linking convening spaces to agenda-setting around equity and misinformation. |

**Table S2d.** *Capacity building and the public health diplomat pipeline*

| *Manuscript claim supported* | *Source* | *Evidence type* | *Specific source-level finding/example* | *Relevance to Section 6* |
| --- | --- | --- | --- | --- |
| Public health diplomacy requires multidisciplinary and cross-sector competencies. | Joshi et al. [3] | PHD summit policy brief | The paper states that strengthening health diplomacy requires multidisciplinary approaches and cross-sector training to prepare professionals to navigate sociopolitical and cultural complexities. | Directly supports the claim that PHD capacity must be intentionally built. |
| PHD requires broad stakeholder engagement, consensus-building, and negotiation. | Joshi et al. [3] | Summit report | The University of Memphis Public Health Diplomacy Lab was created to bring together governments, NGOs, academics, and civil society to improve collaboration and coordination through stakeholder engagement, consensus-building, and negotiations. | Supports the claim that convening spaces can train and enact diplomacy competencies. |
| Health diplomacy training is critical for interdisciplinary competencies. | Joshi et al. [5] | Scoping review | The review states that health diplomacy training is critical for building competencies needed to navigate complex negotiations, cross-cultural engagement, and policy influence. | Supports the “public health diplomat pipeline” argument. |
| Existing health diplomacy education is fragmented, inequitable, and under-evaluated. | Joshi et al. [5] | Scoping review | The authors conclude that health diplomacy education is important for negotiation, leadership, cultural competency, and communication skills, but that current initiatives remain fragmented, inequitable, and under-evaluated. | Supports the claim that unequal access to training/convening opportunities may reinforce capacity gaps. |
| Health diplomacy competencies include negotiation, policy analysis, cross-cultural communication, leadership, and crisis management. | Joshi et al. [5] | Scoping review | Core competencies emphasized across training initiatives included negotiation, diplomacy, cross-cultural communication, leadership, policy analysis, and crisis management. | Directly supports the manuscript’s wording on multidisciplinary competencies. |
| Competency frameworks need to serve students, professionals, diplomats, and health workers. | Joshi et al. [5] | Scoping review | Training initiatives targeted students, professionals, diplomats, health attachés, senior officials, and mixed audiences, using simulations, workshops, curricula, and professional pathways. | Supports the idea that the public health diplomat pipeline spans career stages and institutional locations. |
| Health diplomacy requires coalition-building and advocacy competencies. | Global Health Diplomacy Institutional Network [4] | Competency framework | The framework identifies coalition-building and advocacy as one of five key competencies, emphasizing trust, networks, alliances, and influencing decision-making. | Supports the claim that coalition-building is a learned, formalizable diplomacy skill. |
| Health diplomacy requires cross-cultural communication. | Global Health Diplomacy Institutional Network [4] | Competency framework | The framework includes cross-cultural and interpersonal communication as a core competency, involving collaboration across cultural and political contexts and adaptation to different audiences. | Supports the claim that communication and facilitation are central to the public health diplomat pipeline. |
| Health diplomacy requires political analysis and strategic leadership. | Global Health Diplomacy Institutional Network [4] | Competency framework | The framework includes political analysis/diplomacy skills and strategic decision-making/leadership as core competencies. | Supports the claim that PHD requires more than technical public health expertise. |
| Early-career researchers require conference participation and professional networks. | Herinek et al. [13] | Expert position statement | Herinek et al. recommend connecting ECRs to professional and research networks and supporting attendance and presentations at local, national, and international conferences. | Supports the claim that convening access shapes future capacity and leadership pipelines. |
| EMCRs in LMIC settings need mentorship, networks, funding, and institutional support. | Chikwari et al. [15] | Workshop-based thematic analysis | Chikwari et al. identify lack of individual/institutional support, mentorship, networks, and flawed funding structures as barriers to EMCR leadership in LMIC contexts. | Supports the claim that unequal access limits future diplomatic capacity and reinforces monocultures over time. |
| Systems thinking is a core public health policy capacity. | Silburn [19] | Opinion/review | Silburn argues that systems thinking helps policymakers understand interconnections, feedback loops, stakeholder relationships, unintended consequences, and adaptive policy needs. | Supports the manuscript’s inclusion of systems thinking as a public health diplomacy competency. |
| Systems-informed policy depends on stakeholder coordination and adaptive evaluation. | Silburn [19] | Opinion/review | The paper emphasizes stakeholder coordination, strong governance, continuous monitoring, adaptive feedback loops, and attention to equity, power, and politics. | Supports the claim that PHD capacity involves systems-level thinking, not only communication skills. |
| Whole-of-government and whole-of-society approaches are needed for well-being policy. | WHO [46] | WHO policy pathway document | The WHO framework calls for coordinated, multisectoral approaches, knowledge/resource sharing, local wisdom, lived experience, and whole-of-government/whole-of-society decision-making. | Indirectly supports the capacity-building logic: diplomacy-related public health work requires cross-sector collaboration and inclusive governance capacities. |

**Table S2e.** *Diplomatic monocultures, knowledge hierarchies, and narrowing of policy imagination*

| *Manuscript claim supported* | *Source* | *Evidence type* | *Specific source-level finding/example* | *Relevance to Section 6* |
| --- | --- | --- | --- | --- |
| Global health research is shaped by power asymmetries and knowledge hierarchies. | Kumar et al. [35] | Review / conceptual paper | The paper argues that global health research is sustained by colonial ideas, power asymmetries, and knowledge hierarchies, and that fairer partnerships alone may not shift underlying structures. | Supports the manuscript’s concept of diplomatic monocultures as patterns of epistemic and institutional dominance. |
| Decolonising global health requires shifting decision-making power and redistributing resources. | Kumar et al. [35] | Review / conceptual paper | The authors call for shifting decision-making power, redistributing resources, and holding funders and other power-holders accountable to affected places and people. | Supports the claim that inclusion must change whose priorities shape the agenda. |
| Centralized global governance can impose one-size-fits-all solutions. | De Campos-Rudinsky et al. [36] | Viewpoint | The authors argue that subsidiarity helps avoid one-size-fits-all prescriptions by centralized global organizations and supports contextually adequate solutions. | Supports the claim that narrow policy frames can emerge when local expertise is not meaningfully included. |
| Local communities hold knowledge needed for contextually appropriate responses. | De Campos-Rudinsky et al. [36] | Viewpoint | The authors argue that local communities have deeper understanding of their own circumstances and can contribute valuable knowledge and expertise. | Supports the manuscript’s claim that excluding certain regions or actors narrows policy imagination. |
| Conference and governance design can either broaden or restrict access to global decision-making. | Khorsand et al. [33] | Commentary | The authors argue that virtual WHA formats enabled broader access to voices not typically represented at the conference table, while in-person reconvening risked re-exclusion under unequal vaccine access. | Supports the claim that convening structures can reproduce or mitigate diplomatic monocultures. |
| Whole-of-society infodemic response requires actors beyond usual institutional networks. | Calleja et al. [10] | WHO conference-derived research agenda | The paper argues that health authorities had to build partnerships beyond usual networks, including fact-checkers, media, digital platforms, community organizations, and civil society. | Supports the claim that narrow epistemic communities are less resilient to misinformation shocks. |
| Public health diplomacy should include formal and informal actors. | Joshi et al. [3] | PHD summit policy brief | The authors emphasize the need to combine formal diplomacy with informal diplomacy involving non-state actors. | Supports the manuscript’s framing of conferences as a bridge between formal and informal diplomacy. |
| D&I interventions need to move beyond awareness toward structural outcomes. | Zhao et al. [12] | Meta-review of organizational D&I interventions | Zhao et al. show that D&I interventions vary in strength and outcomes, with diversity training mainly linked to awareness/learning, while structural policy effects are more mixed. | Supports the claim that symbolic inclusion alone may not change underlying monocultures. |
| Tokenism can undermine psychological safety and meaningful participation. | Kataria & Verma [45] | Cross-sectional organizational study | The study reports that perceived tokenism was negatively associated with psychological safety and contextual performance, and argues for moving beyond numerical representation toward inclusive leadership and communication. | Supports the claim that formal inclusion without influence can reinforce rather than disrupt monocultures. |
| Deliberative interaction can reduce or intensify polarization depending on design. | Caluwaerts et al. [44] | Multidisciplinary systematic review | The review finds that communicative encounters can reduce polarization when designed according to deliberative-democracy principles, but can intensify polarization when poorly structured. | Supports the manuscript’s caution that inclusion and consensus-building require careful facilitation. |

**Supplementary Table S3.** *Source-level examples supporting Section 7: Digitalization, hybrid conferences, infodemic governance, and equity-by-design*

**Table S3a.** *Digitalization and hybrid conferences as access-expanding but incomplete equity mechanisms*

| *Manuscript claim supported* | *Source* | *Evidence type* | *Specific source-level finding/example* | *Relevance to Section 7* |
| --- | --- | --- | --- | --- |
| Digitalization and hybrid conferencing can widen access by reducing travel-related barriers. | Doran et al. [29] | Review/guidance article on virtual and hybrid events | Doran et al. state that virtual and hybrid conferences can create opportunities for groups previously excluded from purely in-person formats. They also note that in-person conferences often create barriers through cost, travel, visas, caring responsibilities, disability-related needs, and Global North location patterns. | Supports the opening claim that digital and hybrid conferencing can reduce barriers and widen access. |
| Virtual and hybrid formats are not automatically equitable. | Doran et al. [29] | Review/guidance article | The authors explicitly warn that without effective planning, virtual and hybrid events can replicate biases and exclusions inherent to in-person events. They argue that accessibility and inclusion must be built into pre-event, during-event, and post-event planning. | Directly supports the statement that access alone does not guarantee recognition, visibility, or influence. |
| Including a virtual component as an add-on is insufficient. | Doran et al. [29] | Review/guidance article | Doran et al. argue that it is not enough to include a virtual component at the end; structures should be put in place early to ensure equitable experiences between virtual and in-person participants. | Supports the manuscript’s equity-by-design interpretation of hybrid conferences. |
| Hybrid conferences are preferred by many attendees but require careful design. | Ram et al. [39] | Survey and directed content analysis of conference attendees | In a survey of 823 respondents, 56.9% of those expressing a preference wanted future hybrid formats, 32.0% preferred in-person conferences, and 11.1% preferred virtual conferences. Respondents also differed in what they preferred for in-person versus virtual components. | Supports the claim that hybrid formats have practical relevance but require deliberate design choices rather than a single default model. |
| Hybrid design should distinguish what is best delivered in person versus virtually. | Ram et al. [39] | Survey study | Respondents prioritized clinical skills sessions, expert presentations/discussions, and delegate interaction for in-person components, while virtual components were favored for live-streamed expert presentations, case discussions, updates, and extended access to content. | Supports the idea that hybrid conferences should be designed around specific functions rather than simply streaming an in-person event. |
| Virtual conferences can increase geographic reach and participation from high-burden settings. | Whitfield et al. [30] | Comparative analysis of in-person, virtual, and hybrid TB conferences | The virtual TB conference had 2.5-fold more participants than the average in-person conference, included participants from more than twice as many countries, and attracted 4.5-fold more participants from high-TB-burden countries. | Supports the claim that virtual formats can widen access, especially for geographically underrepresented or high-burden settings. |
| Virtual access can expand participation while maintaining perceived scientific quality. | Whitfield et al. [30] | Comparative conference model analysis | Scientific quality scores for the virtual and hybrid TB meetings were comparable to those of in-person conferences, while virtual participation dramatically reduced estimated CO₂e emissions. | Supports the claim that access-expanding formats need not necessarily reduce perceived scientific quality. |
| Hybrid models can widen access but still preserve HIC dominance in the physical room. | Whitfield et al. [30] | Comparative conference model analysis | In the hybrid TB conference, 91% of in-person participants were from HICs, while virtual participation was geographically broader. | Supports the warning that hybrid formats may widen attendance while leaving in-person visibility and networking concentrated among HIC participants. |
| Virtual events can produce co-benefits for climate and participation. | Yates et al. [28] | Comparative evidence from five international conferences | Moving online reduced travel-related aviation CO₂ emissions and increased attendance, including among attendees from LMICs, without major change in participant ratings. However, online formats reduced participation in social events. | Supports the dual claim that virtual formats can improve access/sustainability but may weaken social/networking dimensions. |
| Virtual formats face challenges related to time zones, digital divides, and reduced informal interaction. | Yates et al. [28] | Comparative conference study | The authors identify challenges for virtual events including emulating intangible in-person interactions, overcoming time-zone limitations, and digital divides. | Supports the manuscript’s claim that digital formats can introduce new forms of gatekeeping. |
| Virtual or hybrid global governance meetings can broaden access to decision-making spaces. | Khorsand et al. [33] | Commentary on World Health Assemblies | Khorsand et al. argue that virtual World Health Assemblies enabled broader access to diverse voices not typically represented at the conference table and reduced cost and visa barriers. | Supports the PHD-specific claim that digital/hybrid convening can widen access to governance-adjacent spaces. |

**Table S3b.** *New gatekeeping mechanisms in hybrid and digital formats*

| *Manuscript claim supported* | *Source* | *Evidence type* | *Specific source-level finding/example* | *Relevance to Section 7* |
| --- | --- | --- | --- | --- |
| Hybrid formats can shift exclusion from travel barriers to time-zone barriers. | Doran et al. [29] | Review/guidance article | Doran et al. identify time zones as a common issue for virtual meetings, requiring planning to make sessions accessible to as many participants as possible and clear communication through time-zone-specific programmes. | Directly supports the phrase “time-zone disadvantages” in Section 7. |
| Platform and app design can create accessibility barriers. | Doran et al. [29] | Review/guidance article | The authors note that conference apps may not be compatible with all devices and can create digital accessibility problems related to colour palettes, fonts, readability, screen readers, and assumptions about device access. | Supports the claim that platform control can affect participation and visibility. |
| Participants need explicit instructions to participate meaningfully in virtual activities. | Doran et al. [29] | Review/guidance article | Doran et al. emphasize that clear instructions on how to submit questions or attend virtual activities and networking opportunities are crucial to avoid inadvertent exclusion. | Supports the claim that interaction opportunities depend on facilitation and platform design. |
| Networking and engagement are difficult to reproduce online. | Doran et al. [29] | Review/guidance article | The authors report recurring difficulties in creating effective networking environments and participant engagement during online meetings; virtual poster sessions were often challenging to design successfully. | Supports the claim that digital attendance does not automatically create network access or coalition-building opportunities. |
| Hybrid events need dedicated networking features to prevent weak online engagement. | Doran et al. [29] | Review/guidance article | The authors recommend group chats, roundtables, matchmaking, and gamified online elements to increase connectivity, especially because online engagement in hybrid events can be low when in-person attendees prioritize physical opportunities. | Supports the need for formats that support meaningful network formation beyond physical co-presence. |
| Remote or virtual participation can suffer from digital fatigue and impersonal interaction. | Moss et al. [27] | Commentary on conferences and collaboration | Moss et al. note commonly cited limitations of virtual interaction, including digital-meeting fatigue, loss of hallway conversations, impersonal interactions, and challenging time zones. | Supports the claim that virtual formats create new participation barriers even when they remove travel barriers. |
| Future conferencing may require mixed or redesigned collaboration models rather than a return to the pre-pandemic norm. | Moss et al. [27] | Commentary | The authors describe post-pandemic possibilities such as alternating in-person and virtual meetings, local hubs linked by internet, or fully virtual formats, while recognizing both benefits and challenges of virtual technologies. | Supports the Section 7 framing of hybrid conference design as an active governance choice. |
| Language barriers in live Q&A can limit equal participation. | Li et al. [41] | Interview study with non-native English-speaking academics | Li et al. show that live Q&A at English-based international conferences requires real-time comprehension and response in a non-native language under stress. They describe the format as spontaneous, time-sensitive, and difficult to prepare for. | Supports the claim that participation design affects who can speak and be recognized. |
| Technological language support may help but is not routinely adopted. | Li et al. [41] | Interview study / design implications | The authors note that tools such as real-time translation and transcription can help alleviate language challenges, but that their adoption remains limited even at HCI conferences. | Supports the argument that inclusive hybrid design requires deliberate language and facilitation support. |
| Q&A interaction can reproduce visibility and recognition inequalities. | Jarvis et al. [40] | Empirical study of Q&A participation | Jarvis et al. describe Q&A participation as highly visible because few people can ask questions; question-asking communicates recognition, authority, and belonging. They found that men disproportionately participated in live Q&A and that women reported more discomfort and fear of backlash. | Supports the claim that access to the session is not the same as comparable opportunity to speak and be recognized. |
| Platform-mediated participation can affect who is “on stage” and who remains peripheral. | Doran et al. [29]; Ram et al. [39]; Li et al. [41]; Jarvis et al. [40] | Cross-source synthesis | Across these sources, participation barriers include time zones, weak networking, inaccessible platforms, limited interaction opportunities, English-language pressure, and gendered Q&A participation. | Supports Section 7’s claim that hybrid formats introduce subtle gatekeeping mechanisms affecting visibility and influence. |
| Digital and hybrid formats can create new access barriers when participants lack reliable internet connectivity, sufficient bandwidth, suitable devices, or technically accessible platforms. | Yates et al. [28]; Doran et al. [29]; Ram et al. [39] | Comparative conference study; review/guidance article; attendee survey | Yates et al. identify digital divides, lower bandwidth and connectivity, fewer IT resources, and internet outages as barriers to equitable online participation. Doran et al. note that virtual and hybrid events may be limited by unreliable internet access due to remote location, poor infrastructure, or financial constraints, and that platform or app design can assume access to compatible devices. Ram et al. report that attendees prioritized technical improvements and stronger interaction opportunities for virtual conference components. | Supports the addition that hybrid equity depends not only on offering remote attendance, but also on reliable connectivity, device access, low-bandwidth-compatible platforms, technical support, and inclusive interaction design. |

**Table S3c.** *Digital health governance and inclusive digital transformation*

| *Manuscript claim supported* | *Source* | *Evidence type* | *Specific source-level finding/example* | *Relevance to Section 7* |
| --- | --- | --- | --- | --- |
| Digital transformation requires governance and inclusive approaches. | WHO [21] | Global strategy document | WHO states that digital health should be ethical, safe, secure, reliable, equitable, and sustainable, and should be developed with transparency, accessibility, scalability, interoperability, privacy, security, and confidentiality. | Directly supports the Section 7 sentence that WHO’s global strategy underscores governance and inclusive approaches in digital transformation. |
| Digital health should support equitable and universal access. | WHO [21] | Global strategy document | The strategy’s vision is to improve health for everyone, everywhere through appropriate, accessible, affordable, scalable, and sustainable person-centric digital health solutions. It states that digital health is valued when it supports equitable and universal access to quality health services. | Supports the claim that digitalization can serve equity goals only when designed around access and inclusion. |
| Digital transformation requires investment in governance, institutional capacity, workforce capacity, and data-use capacity. | WHO [21] | Global strategy document | WHO states that digital health can improve health outcomes if supported by sufficient investment in governance, institutional and workforce capacity, and digital systems/data-use training, planning, and management. | Supports the claim that technology alone is insufficient; digital transformation requires governance infrastructure. |
| Digital health strategies need to be fit for use by countries with limited digital access. | WHO [21] | Global strategy document | WHO states that the strategy is designed for all Member States, including those with limited access to digital technologies, goods, and services. | Supports the manuscript’s equity concern around digital divides and unequal access to digital transformation. |
| Digital technologies create both opportunities and challenges. | WHO [21] | Global strategy document | WHO notes that information and communication technologies present new opportunities and challenges for achieving the Sustainable Development Goals and that digital transformation can be disruptive. | Supports the balanced framing of digitalization as both opportunity and potential gatekeeping mechanism. |
| Least-developed countries face specific barriers to implementing digital health technologies. | WHO [21] | Global strategy document | WHO identifies major impediments including enabling environment, resources, infrastructure, education, human capacity, financial investment, internet connectivity, legacy infrastructure, technology ownership, privacy, security, and adaptation of global standards. | Supports the claim that digital access is uneven and can reproduce inequity if not governed deliberately. |
| Digital health should be people-centred, trust-based, evidence-based, inclusive, equitable, and contextualized. | WHO [21] | Global strategy document | The guiding principles state that digital health should be people-centred, trust-based, evidence-based, effective, efficient, sustainable, inclusive, equitable, and contextualized. | Supports the Section 7 claim that digital and hybrid formats should be treated as equity mechanisms only when deliberately designed as such. |
| Equitable digital systems require coordinated policy pathways. | WHO [46] | WHO policy pathway document | WHO’s well-being policy pathways include “Promoting equitable digital systems,” with specific pathways for digital health equity task forces, equity-based digital skills, equity-oriented data collection, technical solutions for equitable access, and rights-based AI governance. | Additional support for the governance logic of inclusive digital transformation; useful as a supplementary source, not a replacement for [21]. |
| Digital transformation benefits from cross-sector and stakeholder collaboration. | Modlin et al. [8] | Scoping review of equity toolkits for international academic partnerships | Modlin et al. identify recurring toolkit domains such as oversight, partnership dynamics, ethical foundations, contextual factors, procedures/activities, and capacity, with themes including stakeholder engagement, communication, role clarification, funding, and data/product management. | Supports the broader claim that digital and hybrid equity should be governed through structured reflection, communication, and accountability. |

**Table S3d.** *Infodemic governance, trust, communication infrastructure, and credible networks*

| *Manuscript claim supported* | *Source* | *Evidence type* | *Specific source-level finding/example* | *Relevance to Section 7* |
| --- | --- | --- | --- | --- |
| Infodemic management depends on delivering usable information in the right format. | WHO [11] | WHO overview report | WHO states that infodemic management aims to ensure that people have the right information, at the right time, and in the right format, so they can adopt protective behaviours during epidemics. | Supports the statement that communication infrastructures are pivotal during crises. |
| Infodemic management requires social listening, risk communication, misinformation resilience, community engagement, and global conferences. | WHO [11] | WHO overview report | The report’s structure includes social listening, risk communication, resilience to misinformation, community empowerment, high-level/global events, and regional/country approaches. | Supports the claim that infodemic governance depends on trusted networks and communication infrastructures. |
| Infodemics involve uncertainty, distrust, fear, and misinformation. | WHO [11] | WHO overview report | The report describes COVID-19 as accompanied by uncertainty, skepticism, distrust, and fear, creating fertile ground for infodemics. | Supports the claim that trust is central to crisis communication and PHD. |
| Infodemic research agenda-setting can occur through virtual global convenings. | WHO [23] | WHO public health research agenda | The research agenda was the main outcome of the first WHO global infodemiology conference, which took place online between June and July 2020, with follow-up prioritization exercises through October 2020. | Supports the claim that digital convenings can shape shared public health research agendas. |
| Infodemic management requires diverse expertise and global participation. | WHO [23] | WHO research agenda | WHO acknowledges expert panellists, facilitators, and chairs from around the world and from areas such as public health, communication, journalism, digital technology, behavioural science, ethics, and related fields. | Supports the claim that credible networks and diverse expertise are needed during crises. |
| Infodemics can erode trust in health authorities and public health responses. | Calleja et al. [10] | Original paper based on WHO Infodemiology Conference | Calleja et al. define an infodemic as an overabundance of information of variable quality during a public health event, which can lead to confusion, harmful behaviour, and erosion of trust in health authorities and responses. | Directly supports the sentence that trust and credible networks are pivotal in crises. |
| Infodemic response requires partnerships beyond traditional public health actors. | Calleja et al. [10] | WHO conference-derived research agenda | The paper describes infodemic management as requiring partnerships with fact-checkers, media and journalists, digital platforms, community organizations, civil society, and other actors beyond usual public health networks. | Supports the PHD argument that communication networks must be broad and cross-sectoral. |
| Digital health governance includes protection against misinformation and misuse of information. | WHO [21] | Global strategy document | WHO’s guiding principles include protection against misinformation, misuse of information, malicious cyber activities, fraud, exploitation, inappropriate use of health data, racism, and human rights violations. | Links digital health governance directly to infodemic and trust concerns in Section 7. |
| Infodemic work connects digital communication, public trust, and global expert convening. | WHO [11]; WHO [23]; Calleja et al. [10] | Cross-source synthesis | Across these sources, infodemic governance is presented as a networked, multidisciplinary, communication-oriented response involving trusted sources, platforms, communities, and global convenings. | Supports Section 7’s bridge between digitalization, hybrid conferences, infodemic governance, and public health diplomacy. |

**Table S3e.** *Equity-focused hybrid design: scheduling, speaker parity, facilitation, accessible platforms, and network formation*

| *Manuscript claim supported* | *Source* | *Evidence type* | *Specific source-level finding/example* | *Relevance to Section 7* |
| --- | --- | --- | --- | --- |
| Hybrid equity requires planning before, during, and after the event. | Doran et al. [29] | Review/guidance article | Doran et al. organize guidance around three stages: pre-event planning, during-event delivery, and post-event practices. | Directly supports the equity-by-design framing of Section 7. |
| Event design should involve diverse organizers and target-audience analysis. | Doran et al. [29] | Review/guidance article | The authors recommend involving diverse planning teams, including early-career colleagues and historically marginalized groups, and identifying target audiences and their platform needs early in the planning process. | Supports the claim that hybrid equity requires deliberate design, not just technical access. |
| Speaker selection should be broad, meaningful, and avoid tokenism. | Doran et al. [29] | Review/guidance article | Doran et al. recommend aiming for a broad and diverse range of presenters, avoiding tokenism, reaching outside committee networks, and ensuring that diversity does not become a box-ticking exercise. | Supports Section 7’s concern with speaker parity and meaningful visibility. |
| Time-zone accessibility should be designed into the schedule. | Doran et al. [29] | Review/guidance article | The authors recommend assessing where target audiences and speakers are located, considering rerunning sessions, using pre-recorded talks, enabling pre-submitted questions, and providing recordings afterward. | Supports the Section 7 recommendation for deliberate scheduling. |
| Audience questions should be designed for inclusion. | Doran et al. [29] | Review/guidance article | During events, Doran et al. recommend allowing questions verbally or in text, clearly explaining how questions can be asked, monitoring secondary platforms, and considering not recording Q&A to lower barriers. | Supports the claim that inclusive facilitation affects who can speak and be recognized. |
| Technical and staffing support are part of accessibility. | Doran et al. [29] | Review/guidance article | Doran et al. recommend confirming volunteers and staff, clarifying roles, creating communication plans, and ensuring technical support before and during the event. | Supports the argument that hybrid inclusion requires operational infrastructure. |
| Accessibility needs should be collected proactively. | Doran et al. [29] | Review/guidance article | The authors recommend pre-event accessibility questionnaires linked to registration or circulated in advance to identify participant needs and ensure full inclusion. | Supports the claim that accessible platforms and formats require intentional planning. |
| Network formation requires explicit hybrid design features. | Doran et al. [29] | Review/guidance article | Doran et al. recommend group chats, roundtables, matchmaking, gamification, and other online networking features, especially for hybrid events with low online engagement. | Supports the Section 7 phrase “formats that support meaningful network formation beyond physical co-presence.” |
| Speaker parity is necessary because prestige roles are unevenly distributed. | Last et al. [34] | Conference representation analysis | Last et al. show that women and LMIC-affiliated participants can remain underrepresented in prestigious roles, even where overall representation improves. | Supports the claim that comparable speaking and recognition opportunities must be designed intentionally. |
| Invited panels are a concrete site of gendered visibility. | Kamran et al. [47] | Retrospective analysis of invited ASCO panels | Kamran et al. found that 12.1% of invited sessions were manels and that women remained underrepresented in some topics, despite improvements over time. | Supports the claim that who is placed “on stage” remains an equity-relevant design issue. |
| Manels are not neutral; they affect recognition and message quality. | Kibbe & Kapadia [48] | Invited commentary | Kibbe and Kapadia argue that manels limit diversity of perspectives, send harmful messages to junior colleagues, reduce women’s recognition and promotion opportunities, and weaken panel quality. | Supports the claim that speaker parity is part of recognition and influence, not only representation. |
| Q&A participation is part of recognition and authority. | Jarvis et al. [40] | Empirical study | Jarvis et al. state that because few people can ask questions, those who do are highly visible and communicate recognition, authority, and belonging; men disproportionately participated in live Q&A. | Supports the claim that equity-focused facilitation must address who gets heard. |
| Language-supportive tools can improve participation in live Q&A. | Li et al. [41] | Interview study / design implications | Li et al. discuss real-time translation and transcription as tools that can alleviate language-related challenges for non-native English-speaking presenters, while noting limited adoption. | Supports the recommendation for accessible, language-sensitive facilitation and platforms. |
| Equitable hybrid formats should give remote and in-person participants comparable opportunities to speak, interact, network, and be recognized. | Doran et al. [29]; Ram et al. [39]; Jarvis et al. [40]; Li et al. [41] | Cross-source synthesis | Doran et al. support lifecycle accessibility planning; Ram et al. show differentiated attendee preferences for in-person/virtual components; Jarvis et al. show Q&A visibility inequities; Li et al. show language barriers in live Q&A. | Directly supports the concluding claim of Section 7 that digital/hybrid formats become equity mechanisms only when deliberately designed for comparable participation and recognition. |
